# Reproductive isolation and patterns of genetic differentiation in a cryptic butterfly species complex

**DOI:** 10.1111/jeb.12211

**Published:** 2013-08-05

**Authors:** V Dincâ, C Wiklund, V A Lukhtanov, U Kodandaramaiah, K Norén, L Dapporto, N Wahlberg, R Vila, M Friberg

**Affiliations:** * Department of Zoology, Stockholm UniversityStockholm, Sweden; † Institut de Biologia Evolutiva (CSIC-Universitat Pompeu Fabra)Barcelona, Spain; ‡ Department of Karyosystematics, Zoological Institute of Russian Academy of ScienceSt. Petersburg, Russia; § Department of Entomology, St. Petersburg State UniversitySt. Petersburg, Russia; ¶ School of Biology, Indian Institute of Science, Education and Research ThiruvananthapuramThiruvananthapuram, India; ** Department of Biological and Medical Sciences, Oxford Brookes UniversityHeadington, Oxford, UK; †† Laboratory of Genetics, Department of Biology, University of TurkuTurku, Finland; ‡‡ Department of Plant Ecology and Evolution, Evolutionary Biology Centre, EBC, Uppsala UniversityUppsala, Sweden

**Keywords:** courtship, speciation, female mate choice, genetic structure, Lepidoptera: *Leptidea*, species concept

## Abstract

Molecular studies of natural populations are often designed to detect and categorize hidden layers of cryptic diversity, and an emerging pattern suggests that cryptic species are more common and more widely distributed than previously thought. However, these studies are often decoupled from ecological and behavioural studies of species divergence. Thus, the mechanisms by which the cryptic diversity is distributed and maintained across large spatial scales are often unknown. In 1988, it was discovered that the common Eurasian Wood White butterfly consisted of two species (*Leptidea sinapis* and *Leptidea reali*), and the pair became an emerging model for the study of speciation and chromosomal evolution. In 2011, the existence of a third cryptic species (*Leptidea juvernica*) was proposed. This unexpected discovery raises questions about the mechanisms preventing gene flow and about the potential existence of additional species hidden in the complex. Here, we compare patterns of genetic divergence across western Eurasia in an extensive data set of mitochondrial and nuclear DNA sequences with behavioural data on inter- and intraspecific reproductive isolation in courtship experiments. We show that three species exist in accordance with both the phylogenetic and biological species concepts and that additional hidden diversity is unlikely to occur in Europe. The *Leptidea* species are now the best studied cryptic complex of butterflies in Europe and a promising model system for understanding the formation of cryptic species and the roles of local processes, colonization patterns and heterospecific interactions for ecological and evolutionary divergence.

## Introduction

One major revelation stemming from the molecular revolution in biology is that we have long been underestimating the number of species on earth. An increasing number of studies report the presence of cryptic diversity in almost any major taxonomic group under study (Pfenninger & Schwenk, 2007). These examples often come from previously well-defined species that include hidden layers of variation in the form of potential cryptic species that are morphologically indistinguishable, but genetically differentiated (e.g. Knowlton, 1993; Beheregaray & Caccone, 2007; Bickford *et al*., 2007).

The rate of discovery of potential cryptic species has been significantly increased by large-scale DNA sequencing approaches such as DNA barcoding (Hebert *et al*., 2003a,b). So far, however, most studies of cryptic species were solely focused on genetic differences, sometimes even in single genes and over restricted geographical areas with respect to the overall distributions of the considered taxa (e.g. Hebert *et al*., 2004; Hajibabaei *et al*., 2006; Smith *et al*., 2006; Brower, 2010). Furthermore, many of the potential cryptic species are allopatric with respect to their siblings, making the interpretation of genetic differences particularly problematic (Mutanen *et al*., 2012). Thus, whereas there is an increased awareness of the existence and importance of the cryptic fraction of diversity present in nature, there is a general lack of behavioural and ecological studies that address the mechanisms by which this diversity is distributed and maintained across large spatial scales.

One reason for the lack of such comprehensive studies is that carefully examining the ecological and evolutionary background of a putative cryptic species complex is a challenging task. As a first step, systematic and large-scale sampling for molecular analyses is necessary, as well as live material that can be bred and used in behavioural experiments. Therefore, the few examples of cryptic species complexes where the maintenance of reproductive isolation is documented typically come from sympatric populations. These examples include species using long-distance signalling either by acoustic (e.g. Höbel & Gerhardt, 2003; Honda-Sumi, 2005) or by chemical cues (Smadja & Butlin, 2009), where the receiving sex of different cryptic species responds to and navigates towards qualitatively different signals. Another theme of sympatric cryptic species is the elaborate nature of the courtship rituals, such as the courtship signals of several *Drosophila* complexes (e.g. Sawamura & Tomaru, 2002; Yeh *et al*., 2006; Etges *et al*., 2007) or the green lacewings (*Chrysoperla*, Chrysopidae) that require matching male and female courtship songs in order to initiate mating (e.g. Henry *et al*., 1999, 2002). The evolution of premating barriers between incipient and closely related species is predicted to be accelerated in sympatry and leads to reproductive character displacement and assortative mating through reinforcement of mate preferences (Butlin, 1987; Howard, 1993; Liou & Price, 1994; Arnold *et al*., 1996; Servedio & Noor, 2003). These processes generate patterns of stronger premating isolation in sympatry than between allopatric populations of diverging species pairs (Coyne & Orr, 1989, 1997). Interestingly, the opposite pattern can also emerge, if reproductive isolation increases with the geographical distance between populations, either as a by-product of local selection on other traits or as a result of genetic drift (Irwin *et al*., 2001, 2005).

Studies that compare patterns of reproductive isolation within and among sympatric and allopatric populations of newly diverged cryptic species may help to unravel the mechanisms behind species divergence and the maintenance of cryptic variation. Here, we present the results of a comprehensive study on a cryptic species complex of Eurasian butterflies, which has been increasingly promoted as a model for speciation studies. We compare patterns of genetic differentiation at a large geographical scale to experimentally determine patterns of reproductive isolation, between con- and heterospecific individuals of sympatric and allopatric populations of *Leptidea* butterflies. This genus was renowned as one of the first cases of cryptic diversity in butterflies (Réal, 1988; Lorković, 1993; Martin *et al*., 2003) and rapidly became the focus of many studies, but only recently (Dincâ *et al*., 2011a), it has been proposed that it actually consists of a triplet of closely related and morphologically similar species: *Leptidea sinapis* (L., 1758), *Leptidea reali* Reissinger, 1989 and *Leptidea juvernica* Williams, 1946 (see Appendix S1). The finding of what is most likely a new Eurasian butterfly species highlighted the need of revising the conclusions drawn from previous studies of the *Leptidea* system and the importance of sampling the entire distribution of the *Leptidea* complex in a search for additional cryptic diversity.

This study has two major objectives. First, we perform a geographically comprehensive sampling effort and report molecular data on genetic relationships and differentiation in the *Leptidea* species complex using both mitochondrial and nuclear DNA markers with specimens distributed over the entire range of the species complex (Dincâ *et al*., 2011a). We thereby search for additional cryptic variation and identify target populations of interest for our second major objective – that of performing behavioural experiments testing reproductive isolation among sympatric and allopatric populations of the three species. We find that the three species are strongly reproductively isolated through female choice of conspecific males but that no such isolation occurs between geographically distant populations within species. Finally, we show that, at least in Europe, the discovery of additional entities hiding within the currently acknowledged triplet of cryptic species is unlikely.

## Materials and methods

### Molecular analyses

**Figure 1:**
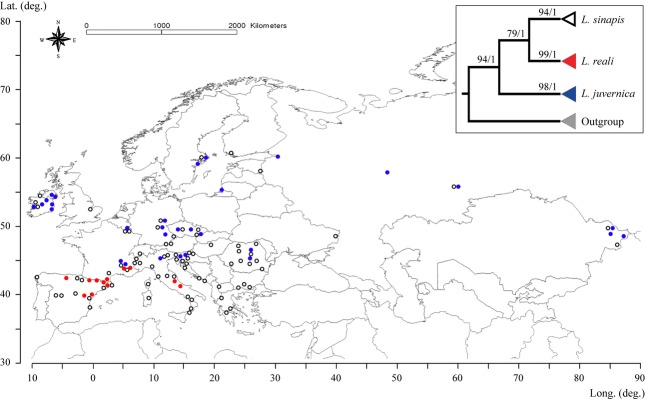
Map of Europe and north-western Asia showing the sample locations of sequenced *Leptidea sinapis* (white circles), *Leptidea reali* (red circles) and *Leptidea juvernica* (blue circles) used in this study. The upper right corner illustrates the relationships between the three species as inferred by the molecular markers used in this study (COI and ITS2). Maximum-likelihood bootstrap supports (≥ 50) and Bayesian posterior probabilities (≥ 0.5) are shown next to recovered nodes and represent the output of the analyses of the combined data set of COI and ITS2 (see Fig. S3).

The analyses were based on both mitochondrial (418 COI sequences) and nuclear (173 ITS2 sequences) DNA markers. The majority of specimens were obtained through field sampling by the authors and by collaborators from different parts of western Eurasia (Fig.1; see Acknowledgments, Table S1). In addition, already published *Leptidea* COI sequences available in GenBank that overlapped our fragment by at least 620 base pairs (bp) were added to the data set (Table S1). The detailed PCR and sequencing conditions can be found in Appendix S1.

The 292 sequences (199 COI and 93 ITS2) obtained in this study have been submitted to GenBank (Table S1). Sequences were aligned using geneious pro 4.7.5 (Drummond *et al*., 2009). For COI, the 658-bp-long alignment comprised 410 specimens of *L*. *sinapis* (236 samples), *L. reali* (61) and *L. juvernica* (113) and consisted exclusively of sequences longer than 620 bp. For ITS2, the 715-bp-long alignment comprised 169 sequences of *L*. *sinapis* (88), *L. reali* (25) and *L. juvernica* (66) and consisted exclusively of sequences longer than 615 bp.

A total of 45 (COI) and eight (ITS2) unique haplotypes of *L. sinapis*,*L. reali* and *L. juvernica* were obtained using the program tcs 1.21 (Clement *et al*., 2000). Maximum parsimony haplotype networks were constructed for each marker using tcs 1.21, with a 95% connection limit. The COI network presented four loops (all in *L. sinapis*), which were broken according to frequency and geographical criteria (Excoffier & Langaney, 1989).

### Phylogenetic inference

Maximum-likelihood (ML) and Bayesian inference (BI) analyses were run for each marker separately, as well as for the combined data set. The ML phylogenetic trees were inferred using phyml 2.4.4 (Guindon & Gascuel, 2003) and implemented in geneious pro 4.7.5 (Drummond *et al*., 2009). The BI analyses were run with beast 1.6.2 (Drummond & Rambaut, 2007). For specifics on assumptions and model selections, see Appendix S1.

### Mapping genetic divergence

**Figure 2:**
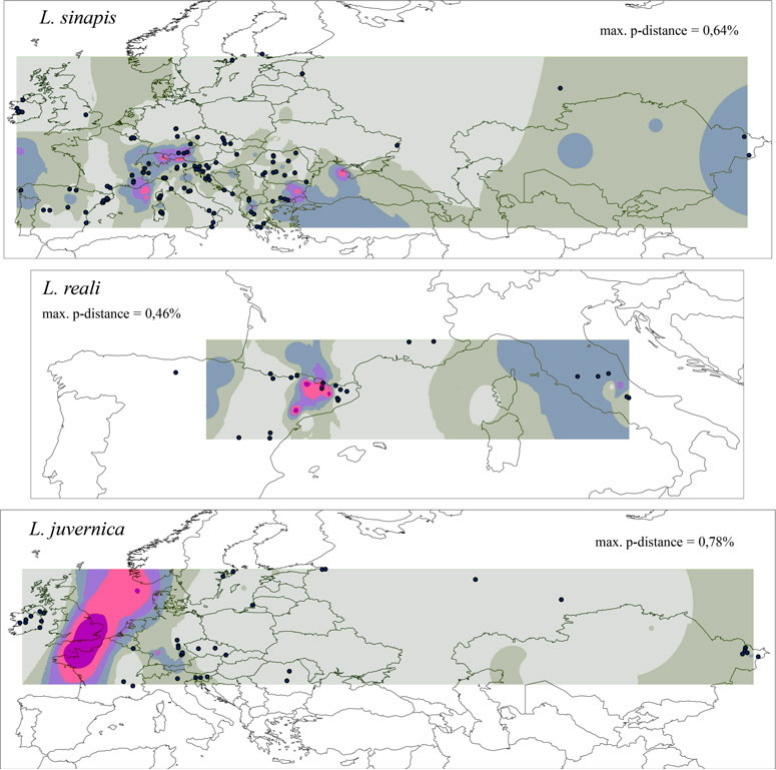
Maps of genetic divergence for *Leptidea sinapis*,*Leptidea reali* and *Leptidea juvernica* based on COI uncorrected p-distances. The colours indicate increasing levels of genetic divergence in the following order: light grey, grey, light blue, light violet, pink and violet. All colours indicate geographical areas situated midway between different haplotypes.

For each DNA marker (COI and ITS2), uncorrected p-distances between all specimens within each species were used to generate genetic divergence maps as a graphical overview of the distribution of intraspecific genetic variability across the studied area (Fig.2). For each species and marker, a matrix of p-distances and a table of GPS coordinates of the corresponding samples were imported in r (2.14.0) with the library deldir installed. We calculated a Delaunay triangulation among GPS coordinates for the collection sites. The midpoints of segments composing the Delaunay triangulation were identified, and the p-distance between the pair of sites composing each segment was attributed to the midpoints. Midpoints and their p-distance were imported in arcmap10 by Esri (http://www.esri.com), and the p-distance values interpolated through inverse distance weighting using the Spatial Analyst tool. For more details, see Appendix S1.

### Courtship experiments

The courtship experiments were performed at the Department of Zoology, Stockholm University with laboratory-reared *Leptidea* specimens originating from wild-caught females from different parts of Europe and Asia. The species affiliation of the females that laid the eggs was confirmed *post-mortem* through genitalia examination and/or DNA sequencing. For details about the laboratory rearing conditions, see Appendix S1. Data were collected over a total of 6 years (2004–2006, 2010–2012). The laboratory populations included *L. sinapis* from Spain (Catalonia, El Brull, Montseny area, where *L. sinapis* and *L. reali* occur in sympatry) and Sweden (Riala, approximately 40 km north of Stockholm, where *L. sinapis* and *L. juvernica* occur in sympatry); *L. reali* from Spain (Catalonia, El Brull, Montseny area, where *L. sinapis* and *L. reali* occur in sympatry); and *L. juvernica* from Sweden (Kronängen, approximately 65 km south-west of Stockholm, where *L. sinapis* and *L. juvernica* occur in sympatry), eastern Kazakhstan (sites situated between 30 and 80 km south of Zyryanovsk, where *L. sinapis* and *L. juvernica* occur in sympatry) and Ireland (sites in Cork County, where *L. juvernica* is allopatric with respect to *L. sinapis*).

Data on reproductive isolation within and between the genetic complexes were tested with two different data sets. We first performed a reciprocally balanced analysis of patterns of reproductive isolation between the three cryptic species, including exclusively *L. sinapis* and *L. reali* from Spain, and *L. juvernica* from Sweden. Females of all species were presented to males of all species and included 118 courtship trials of which 52 involved conspecific pairs and 66 represented males courting heterospecific females. To determine patterns of reproductive isolation also at the within-species level, we then generated a data set including all conspecific courtship trials (*n* = 307) between *L. sinapis* butterflies of the same and of different populations (samples from Spain and Sweden) and between *L. juvernica* butterflies of the same and of different populations (samples from Sweden, Ireland and Kazakhstan) collected over the course of 6 years. The results from 89 of these interactions were previously reported in the study by Friberg *et al*. (2008a).

During the elaborate courtships, a *Leptidea* male sits opposite to a female while oscillating his proboscis in front of her. A female can signal mating acceptance by making her abdomen accessible for male copulation attempts, but seems unable to reject the male. This means that unsuccessful courtship attempts are not terminated until the male aborts the display and flies away (for a detailed description of the courtship, see Friberg *et al*., 2008a; and Appendix S1). The courtship trials were performed in accordance with the protocol developed in the study by Friberg *et al*. (2008a). Virgin females were presented to nonmated males in individual cages, and we measured the courtship duration until the female accepted mating (female acceptance time). Alternatively, when mating was not successful, we recorded the time until the male terminated courtship (male giving-up time). Each individual was used only once, with the exception of a few males of varied origin that were used again (not in the same day) due to a shortage of specimens. The same female never met the same male so all male–female interactions were unique.

All courtship data were analysed in the statistical program r (2.14.0; R Development Core Team, 2011). Data on courtship duration (female acceptance time/male giving-up time) were log-transformed prior to the analysis to meet the assumptions of linear modelling (anova II). Binomially distributed response variables were tested in logistic regressions, with logit as link function. To all models including data from butterflies of different generations (spring/summer), we added this category as a block factor, because at least *L. juvernica* butterflies of the different generations tend to differ in mating propensity when reared under the same conditions (Friberg & Wiklund, 2007).

In the models testing for reproductive isolation between the three species (*L. sinapis* and *L. reali* from Spain and *L. juvernica* from Sweden), we tested the female preference (yes/no) in a logistic regression with male and female species and their interaction as categorical predictor variables. Female acceptance time of successful courtships was tested in a linear model with species as categorical predictor, whereas the male giving-up time of unsuccessful courtships was tested in a linear model with male and female species and their interaction as categorical predictor variables.

In the data set focused on conspecific courtships of *L. sinapis* and *L. juvernica*, we tested the female preference (mating yes/no) for accepting mating in a logistic regression with the species (*L. sinapis*/*L. juvernica*), the geographical status (males from allopatric or sympatric populations) and their interaction as factors. We also tested whether the time to female mating acceptance differed between allopatric and sympatric trials in a linear model with butterfly species, the geographical relationship between the male and the female and their interactive effect as categorical predictors. Courtship experiments data were deposited in the Dryad repository: doi:10.5061/dryad.5b79m.

## Results

### Molecular data

Both single-marker and combined analyses based on the mitochondrial (COI) and nuclear (ITS2) markers recovered three well-defined monophyletic groups corresponding to *L. sinapis*,*L. reali* and *L. juvernica* (Fig.1 and Fig. S1–S3). Relationships among these clades were well resolved (especially in the Bayesian analysis) and *L. sinapis* appeared as sister to *L. reali*, whereas *L. juvernica* was sister to the other two, in concordance with previous findings (Dincâ *et al*., 2011a). Species determination based on genital morphology was always congruent with the results from both markers, although it should be noted that the genitalia only allow identification of two groups: one corresponds to *L. sinapis* and the other comprises *L. reali* plus *L. juvernica*, whose genitalia are apparently indistinguishable (Dincâ *et al*., 2011a).

The genetic data showed that *L. sinapis* is widespread from Ireland in the north-west to at least Spain, Italy and Greece in the south (including the islands of Sardinia and Corsica), the Nordic countries and Russia in the north and eastern Kazakhstan in the east. *Leptidea reali* is limited to northern Iberian Peninsula, southern France and Italy and does not show any overlap with *L. juvernica*, which is widespread in the remaining parts of the joint distribution with *L. sinapis* (Fig.1). Intraspecific genetic variation was low given the geographical area covered: in the case of COI, the maximum uncorrected p-distance was 0.76% (five substitutions) for *L. sinapis*, 0.46% (3 substitutions) for *L. reali* and 0.76% (5 substitutions) for *L. juvernica*. The 658 bp of the COI gene sequenced showed a minimum of 2.28% uncorrected p-distance (15 substitutions) between *L. sinapis* and *L. juvernica*, whereas *L. reali* and *L. juvernica* displayed a minimum of 1.82% uncorrected p-distance (12 substitutions). The sister species *L. sinapis* and *L. reali* displayed a minimum uncorrected p-distance of only 0.76% (five substitutions).

**Figure 3:**
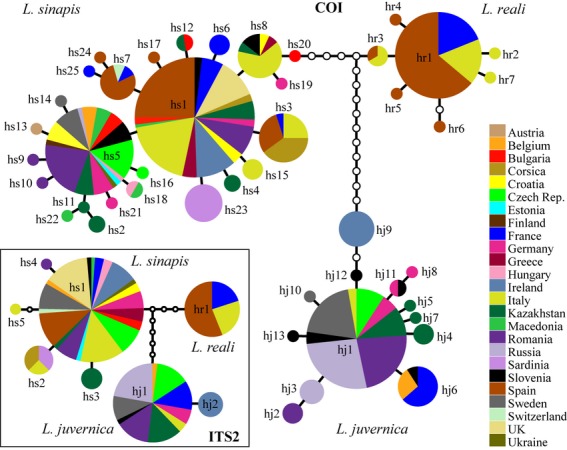
Statistical parsimony COI haplotype network inferred from 410 specimens of *Leptidea sinapis*,*Leptidea reali* and *Leptidea juvernica*. Circle areas are scaled to represent each haplotype's relative frequency in the total sample. Each branch represents one point mutational step, and small white circles represent ‘missing’ haplotypes. The haplotype network based on 169 ITS2 sequences is embedded in the bottom left corner.

Based on COI, *Leptidea sinapis* displayed remarkable genetic homogeneity, with the most notable areas of (albeit very slight) differentiation present in the Alps and, to a lesser extent, in the Balkans (Fig.2). The ITS2 data showed that Corsica and Sardinia were slightly differentiated from most mainland areas and eastern Kazakhstan also displayed a small degree of differentiation (Fig. S4). *Leptidea reali* displayed local COI differentiation across Catalonia (north-eastern Spain) and also a slight separation in central Italy, due to the presence of several haplotypes differing from the most common variant (Figs2 and 3). The nuclear marker ITS2 did not display any divergence in *L. reali* because all samples analysed shared the same haplotype, and an ITS2 genetic distance map was not generated in this case. The COI data set for *L. juvernica* showed that the most notable pattern occurred between the Irish population and all the mainland sites (Figs2 and 3). The ITS2-based map indicated the same separation of Ireland, whereas the continental areas lacked differentiation (Fig. S4).

The *L. sinapis* specimens could be grouped into 25 different COI haplotypes inferred from 236 specimens (Fig.3). The COI haplotype variation within *L. sinapis* was dominated by two common and widespread haplotypes. The most common haplotype (hs1) was widely spread, from Ireland in the west, to Spain and Greece in the south and to Kazakhstan in the east. The second most common haplotype (hs5) was also present in Kazakhstan, as well as in northern and central Europe (Fig.3). The only haplotype specific to a region was that of the nine Sardinian specimens sequenced (hs23), which included a single-point mutation compared with the most common haplotype (hs1; Fig.3). Genetic variability was considerably lower in ITS2, because only five different haplotypes were identified among 88 specimens analysed. One haplotype was by far the most common (hs1), with the others being represented by few specimens (Fig.3). The *L. sinapis* specimens from eastern Kazakhstan had slightly different ITS2 haplotypes, and specimens from Corsica and Sardinia had identical ITS2 sequences (hs2) and appeared somewhat differentiated from mainland, although two samples from north-eastern Italy also displayed this haplotype (Fig.3).

Seven COI haplotypes (inferred from 61 specimens) and a unique ITS2 haplotype (based on 25 specimens) were detected in *Leptidea reali*. The distribution of this species seems to be limited to northern Iberian Peninsula, southern France and Italy, with one major COI haplotype shared by all areas and with six satellite variants (Fig.3).

*Leptidea juvernica* displayed 13 COI haplotypes based on 113 sequenced samples. Most of them belonged to one widespread haplotype (hj1), but it is worth noting that all nine Irish individuals shared the same haplotype (hj9), which differed in two base pair substitutions from the closest European variant (Fig.3). A similar pattern was observed based on ITS2 haplotypes (inferred from 56 samples), where one common haplotype was shared by all mainland regions sampled (hj1), whereas the second one was restricted to Ireland (Fig.3). The Irish *L. juvernica* thus represent a distinct lineage that is sister to all other populations of *L. juvernica*.

### Courtship experiments

**Figure 4:**
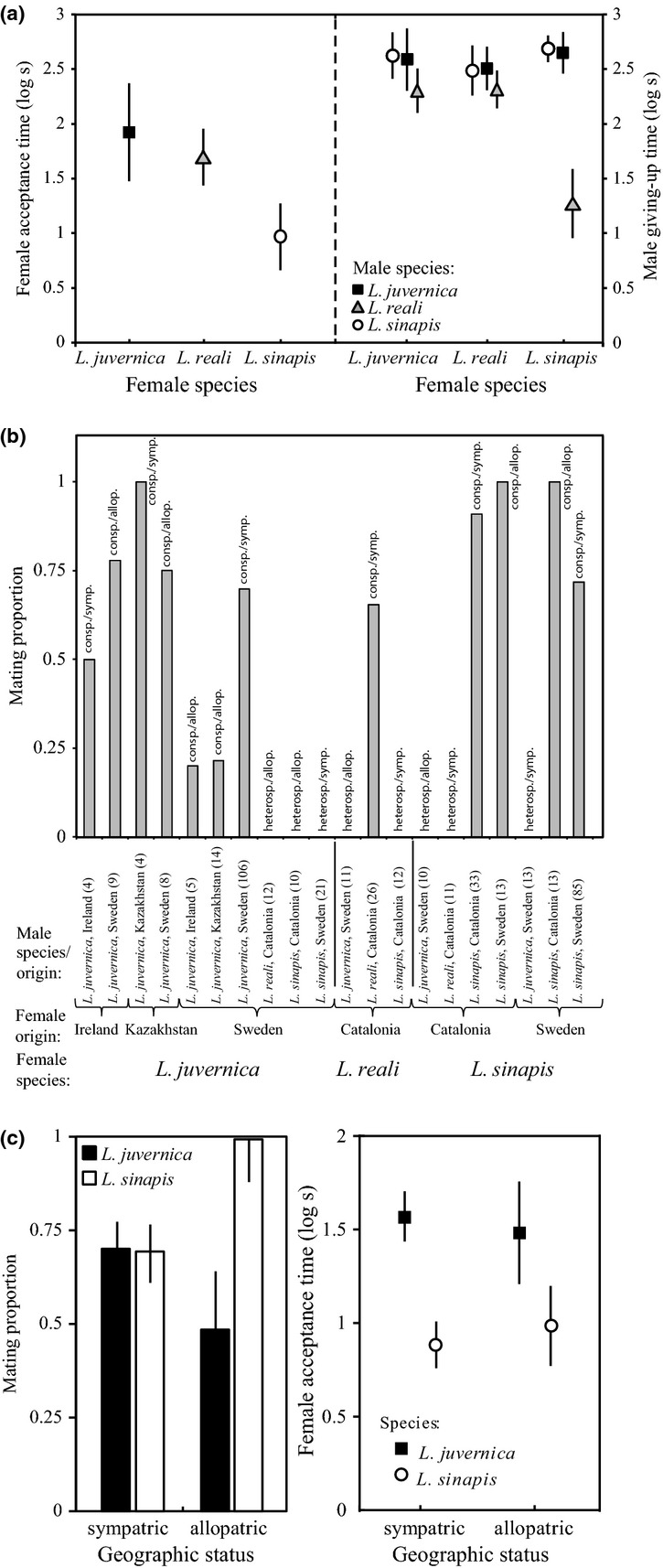
(a) Average female time to acceptance of successful (conspecific) courtships (log s ± 95% CI; left panel) and the average male giving-up time of unsuccessful con- and heterospecific courtships (log s ± 95% CI; right panel) for trials between the core populations of the three species (*Leptidea sinapis* and *Leptidea reali* from Spain and *Leptidea juvernica* from Sweden); (b) the outcome of mating trials between all different populations and species in this study expressed as the proportion of trials that resulted in mating in each specific male–female combination. The species relationship (heterospecific/conspecific) and the geographical status (allopatric/sympatric) are indicated above each bar, and the number of courtship trials is given in parentheses above each population combination; (c) the proportion of conspecific trials in the larger *L. sinapis* and *L. juvernica* data set that resulted in female mating acceptance depending on whether the couple descended from the same or different populations (left panel), and the female acceptance time of successful courtships (log s ± 95% CI) depending on whether females were courted by local or nonlocal males (right panel).

No heterospecific courtship resulted in mating, whereas the intraspecific courtships that served as control ended in mating in 67% (35) of the cases (Fisher's exact *P *<* *0.001; Table1). The overall probability for mating acceptance did not vary with male (logistic regression: 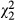 = 0.87, *P = *0.65) or female species (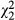 = 1.03, *P = *0.60), whereas the assortative mating expressed by all species was indicated by a highly significant interaction term (male species × female species: 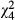 = 76.3, *P *<* *0.001). On average, the 10 *L. sinapis* females that accepted mating needed 16 s to initiate mating (±SD 16 s), which was significantly faster than the 17 *L. reali* (88 s ± 99 s) and the 8 *L. juvernica* females (171 s ± 178 s) that accepted the conspecific male courtship (anova II female species *F*_2,32_ = 7.67, *P *=* *0.0020; Fig.4a). The average unsuccessful courtship was of similar length regardless of whether males courted con- or heterospecific females, with one important exception: the average giving-up time of *L. reali* males courting *L. sinapis* females was significantly shorter (38 s ± 55 s) than that of any other courtship combination (all other average giving-up times > 242 s; male species *F*_2,74_ = 26.5, *P *<* *0.001; female species *F*_2,74_ = 10.5, *P *<* *0.001; male species × female species *F*_4,74_ = 9.6, *P *<* *0.001; Fig.4a). Both *L. sinapis* and *L. reali* males incorporated intermittent wing strokes in the courtship ritual in courtship bouts that lasted longer than 20–30 s, which was never observed for male *L. juvernica* (cf. Friberg *et al*., 2008a).

**Table 1:** The outcome of the reciprocal mating presentations between males and females of the core populations (*Leptidea juvernica* from Sweden, *Leptidea reali* and *Leptidea sinapis* from Spain; number of matings/number of trials). Conspecific interactions are highlighted in bold font

Males	Females		
	*L. juvernica* (Sweden)	*L. reali* (Spain)	*L. sinapis* (Spain)
*L. juvernica* (Sweden)	**8/13**	0/11	0/10
*L. reali* (Spain)	0/12	**17/26**	0/11
*L. sinapis* (Spain)	0/10	0/12	**10/13**

In the larger data set, a total of 69% (91 of 131) of the *L. sinapis* females and 70% (74 of 106) of the *L. juvernica* females accepted courtships from sympatric conspecifics. All 26 courtships between allopatric Swedish and Spanish *L. sinapis* ended with female mating acceptance, and 47% (17 of 36) interpopulation courtships between *L. juvernica* from Sweden and Kazakhstan and between Sweden and Ireland ended with mating acceptance (logistic regression: generation 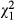 = 2.71, *P *=* *0.10; species 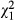 = 4.37, *P *=* *0.037; geographical status 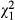 = 0.1, *P *=* *0.74; Species*Geographical status 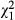 = 23.0, *P *<* *0.001; Fig.4b).

In the interpopulation courtship of *L. juvernica*, Swedish females were especially reluctant to accept males from allopatric populations (Fig.4), but the sample sizes were too low to further test for any asymmetric isolation patterns. Again, females of *L. sinapis* accepted mating after a shorter courtship duration (19 ± 24 s) than *L. juvernica* females (90 ± 131 s), and they did so regardless of being courted by sympatric or allopatric males (linear model anova II: generation *F*_1,209_ = 0.12, *P *=* *0.73; species *F*_1,209_ = 64.7, *P *<* *0.001; geographical status *F*_1,209_ = 0.063, *P *=* *0.80; Species*Geographical status *F*_1,209_ = 0.31, *P *=* *0.34; Fig.4c, Fig. S5). Unsuccessful conspecific courtships of *L. sinapis* and *L. juvernica* lasted as long regardless of whether they were performed for females from allopatric or sympatric populations (linear regression anova II: generation *F*_1,81_ = 0.25, *P *=* *0.88; species: *F*_1,81_ = 1.75, *P *=* *0.19; geographical status *F*_1,81_ = 0.83, *P *=* *0.37; Fig. S5; note that in this analysis, no giving-up time was available for allopatric *L. sinapis*, because all these courtships were accepted by the females).

## Discussion

*Leptidea sinapis*,*L. reali* and *L. juvernica* are reproductively isolated. Not a single of a total of 66 heterospecific courtships between Swedish *L. juvernica*, Spanish *L. sinapis* and Spanish *L. reali* resulted in mating. By contrast, matings occurred between all combinations of conspecific populations, even when these were geographically very distant, such as Spanish and Swedish *L. sinapis* (approximately 2200 km), Kazakhstani and Swedish (approximately 4300 km) and Irish and Swedish *L. juvernica* (approximately 1800 km; Fig.4), and we can therefore conclude that there is no strong distance effect on mating isolation. Thus, our results discard the hypothesis that the Swedish *L. juvernica* and the Spanish *L. reali* populations would represent two isolated ends of a continuum of populations that are not isolated from adjacent populations (c.f. a ring species; e.g. Irwin *et al*., 2001, 2005). The perfect congruence between the patterns of reproductive isolation emerging from the courtship data set and the three well-defined genetic clusters first presented by Dincâ *et al*. (2011a) and further supported by this study proves beyond doubt the species status of *L. sinapis*,*L. reali* and *L. juvernica*.

The extensive geographical sampling clarifies to a considerable extent the distribution of the three species in Europe (Fig.1). *Leptidea sinapis* is widespread across the studied area and can sometimes cohabitate with either *L. reali* or *L. juvernica*. The latter two species have not yet been found in sympatry, whereas *L. reali* is restricted to the western Mediterranean, *L. juvernica* is widely distributed from Ireland to eastern Kazakhstan (Fig.1). The closest documented populations of the latter two species are separated by approximately 90 km in an area in south-eastern France (Dincâ *et al*., 2011a). France and Italy are currently the only countries known to have all three species in their fauna and may be particularly suitable targets for more detailed studies of potential contact areas. Our results confirm that *L. reali* does not occur outside the Iberian Peninsula, southern France and Italy, and previous results on the ecology of *Leptidea* performed on populations outside these areas can thus now be attributed to the pair *L. sinapis* and *L. juvernica* (e.g. Freese & Fiedler, 2002; Beneš *et al*., 2003; Friberg & Wiklund, 2007, 2009, 2010; Friberg *et al*., 2008a,2008b,c, 2013; Nelson *et al*., 2011; Sachanowicz *et al*., 2011).

The combined use of mitochondrial and nuclear markers allowed detection of any potential F1 hybrids or introgression between any of the three species; however, none were detected. The lack of such cases, together with the fact that not a single one of the heterospecific courtships resulted in female mating acceptance, suggests that hybridization is, at most, an uncommon event. The mapping of genetic diversity revealed very low levels of genetic variation also between geographically distant samples (Fig.2, Fig. S4). As expected, COI displayed more genetic variability compared with ITS2, due to a higher mutation rate and the lower effective population size of the mitochondrial DNA marker.

The most striking pattern detected involved the Irish samples of *L. juvernica*, which were differentiated from all other populations studied based on both COI and ITS2. In this context, the courtship experiments were important for determining that this population is not yet another cryptic species. Indeed, there is no reproductive barrier between Irish and continental *L. juvernica* and the former thus represent a distinct lineage within this species. The resulting offspring of these crosses were reared to pupation and did not show any reduced viability compared with within-population crosses (data not shown), which further emphasizes the existence of interpopulation compatibility. However, the results also suggest that allopatric *L. juvernica* courtships less often led to mating than sympatric courtships (Fig.4b), whereas all conspecific trials between allopatric *L. sinapis* from the Swedish and Spanish laboratory populations resulted in female mating acceptance. Potentially, these results reflect an influence of local selection on female mating preference in these populations of *L. juvernica*. Female mating propensity could be under especially strong selection in areas where a species is in the local minority (cf. Noriyuki *et al*., 2012; Friberg *et al*., 2013), and this hypothesis is tentatively supported by the observation that the reduced female mating acceptance of allopatric conspecific males was especially pronounced among Swedish *L. juvernica* females. In Sweden, *L. juvernica* is a habitat specialist with a patchy distribution on meadows surrounded by woodland and therefore virtually always occurring in sympatry with *L. sinapis*, which is a habitat generalist in this area (Friberg *et al*., 2008b,2008c). However, the sample sizes on allopatric mating preferences are too moderate to draw any strong conclusions about the causes and relevance of this pattern. Future studies will have to determine to what extent local selection on female mating preference from heterospecific courtship interference also can affect patterns of within-species reproductive isolation between allopatric populations.

Patterns of weak genetic diversification occurred over narrow areas in correspondence with sea straits separating the most isolated islands (Ireland, Sardinia and Corsica) and with the highest mountain barriers (Alps and Pyrenees). The lack of major genetic variation across the continental areas is perfectly congruent with the mating results showing no reproductive barriers between distant populations of the same species. Such a concordance among different analyses suggests a high homogeneity among conspecific populations, which seems to only be interrupted by major geographical barriers. Thus, the current data indicate that, at least in Europe, additional layers of cryptic diversity are unlikely to be found within the *Leptidea* triplet.

The intraspecific genetic structures reported here are even more homogeneous than expected given their recent estimated origin of approximately 270 000 years for the triplet (Dincâ *et al*., 2011a). This suggests that, at least for *L. sinapis*,*L. reali* and continental *L. juvernica*, current distributions are the result of a post-glacial colonization from a single glacial refugium for each species: no trace of differentiated intraspecific lineages was detected. Only the Irish lineage of *L. juvernica* represents an interesting exception. Its genetic divergence suggests that populations of this species may have survived in southern Ireland during the last glacial maximum. Indeed, parts of this region were apparently not glaciated (Yalden, 1999; Knight, 2004) and have been hypothesized as a glacial refugium for other taxa (e.g. Chevolot *et al*., 2006; Hughes *et al*., 2006; Hoarau *et al*., 2007; Teacher *et al*., 2009). Alternatively, post-glacial population dynamics may have allowed the colonization of Ireland by this lineage followed by its extinction from any other locations, as suggested to have occurred for many insular endemic butterflies (Dapporto, 2010a).

The overall distribution of the three species, with *L. sinapis* being sympatric with both its siblings and *L. reali* and *L. juvernica* being allopatric, is another interesting aspect. A recent study on *L. sinapis* and *L. juvernica* suggests that heterospecific sexual interference manifested by the male inability to distinguish con- from heterospecific females can generate severe costs of being in the local minority and select for habitat isolation (Friberg *et al*., 2013). Similar mating costs of being in the local minority have been reported from two sibling species of predatory ladybirds, *Harmonia axyridis* and *H. yedoensis*, where the latter species is negatively affected by living in local sympatry with its close relative, due to costs involved in heterospecific courtships (Noriyuki *et al*., 2012). The heterospecific courtships might thus set a limit to how many species can coexist in an area, which provides one possible explanation to why *L. juvernica* and *L. reali* have not yet colonized each other's ranges.

Alternatively, selection could also favour male species recognition. In this study, we find a potential indication of such local selection on *L. reali* males, which courted sympatric *L. sinapis* females for consistently shorter time than their conspecific females, or the *L. juvernica* females (Fig.4a). This pattern could potentially reflect a local reinforcement of mate preferences among *L. reali* males to avoid courting heterospecific females, although future studies are warranted for determining the generality of this pattern, and whether the *L. reali* males are unwilling to court also nonallopatric *L. sinapis*.

Cryptic species are often found to coexist (Bickford *et al*., 2007), and this pattern raises questions about the origin of species and about the potential for cryptic species to become examples of reproductive isolation having evolved in sympatry (but see McBride *et al*., 2009). Many studies on cryptic species, however, are restricted to DNA barcoding data of a set of individuals from a geographically limited area, and less is known about the *raison d'être* for their genetic isolation and to what extent the cryptic species are ecologically and geographically diverged (Thompson, 2008; McBride *et al*., 2009). In some cases, there is evidence suggesting ecological divergence between cryptic species (e.g. Amiet, 1997; but see McBride *et al*., 2009; or Hebert *et al*., 2004; but see Brower, 2010), but the patterns of ecological divergence in cryptic species have rarely been linked to the actual pattern of reproductive isolation in heterospecific courtships (Funk *et al*., 2002).

Furthermore, genetic differences found in allopatry do little to prove species status, unless they are corroborated with mating experiments, and, in relevant cases, studies of hybrid viability. Such experiments are usually difficult to perform and are lacking for the vast majority of potential cryptic taxa with allopatric distributions. Thus, many cryptic species defined solely on genetic data can be questioned under a critical scrutiny. In this context, the *Leptidea* triplet represents a good and rare example of a case documented not only based on genetic and morphological data, but also on extensive mating experiments. For a comparison, none of the recent discoveries of new cryptic butterfly species in Europe has included tests of reproductive isolation (e.g. Kolev, 2005; Nazari & Sperling, 2007; Dapporto, 2010b; Dincâ *et al*., 2011b).

This study has demonstrated a strong correlation between premating reproductive isolation and patterns of genetic variation among the cryptic species *L. sinapis*,*L. reali* and *L. juvernica*. The extensive genetic data set examined for Europe indicates that it is unlikely that additional cryptic species are hidden within the currently known triplet. Moreover, it represents a unique case in European butterflies involving a triplet of cryptic species documented by combining molecular, morphological and behavioural data on reproductive isolation within and among species and thereby represents a step forward not only for the development of this model system, but also for the study of cryptic species in general.

## Supporting Information

Appendix S1 Description of the study system and supplementary materials and methods (fully referenced).

Table S1 Individual sample data and GenBank accession numbers.

Figure S1–S5 Phylogenetic trees for COI, ITS2 and COI + ITS2, mapping of genetic divergence for ITS2 and supplementary courtship results.
