# Supplementary material for: Reproductive isolation and patterns of genetic differentiation in a cryptic butterfly species complex
Source: J Evol Biol. 2013 Aug 5;26(10):2095–106. doi: 10.1111/jeb.12211 (PMC4413813; doi:10.1111/jeb.12211)
Supplement: Appendix S1 [file jeb0026-2095-sd1.docx]

**Appendix S1**

**Reproductive isolation and patterns of genetic differentiation in a cryptic butterfly species complex**

Vlad Dincă, Christer Wiklund, Vladimir A. Lukhtanov, Ullasa Kodandaramaiah, Karin Norén, Leonardo Dapporto, Niklas Wahlberg, Roger Vila, Magne Friberg

**The study system**

European butterflies are arguably the best studied group of invertebrates in the world and the recent discovery of complex layers of cryptic diversity in *Leptidea* represented unexpected events. *Leptidea sinapis* (the Wood White), was described by Carl von Linné in Sweden in 1758. Only 230 years later researchers realized that a second species, *Leptidea reali* Reissinger, 1989 (Real´s Wood White), was “hidden” within the common and widespread *L. sinapis* (Réal 1988; Lorković 1993). The two species had identical wing morphology, but have been shown to be separable based on genitalia and DNA markers (e.g. Lorković 1993, Fumi 2008; Martin *et al*. 2003). At this point, the species pair *L. sinapis - L. reali* became the subject of an increasing number of studies dealing with their distribution, ecology and behaviour so that it has become an emerging model to study speciation among cryptic species. The value of the system has been increased by the recent discovery that *L. sinapis* is the metazoan organism with the highest known chromosome number variability ranging from 2n=56 in eastern Kazakhstan to 2n=106 in north-eastern Spain, in a longitudinally-oriented cline (Lukhtanov *et al*. 2011). Interestingly, a recent study combining genetic (mitochondrial and nuclear DNA markers) with karyological and morphological data revealed the presence of yet another cryptic genetic entity hidden within the range of *L. reali* (Dincă *et al*. 2011). The new entity was referred to as *Leptidea juvernica* Williams, 1946 (Cryptic Wood White) and is the sister taxon to *L. sinapis* + *L. reali* (Dincă *et al*. 2011).

**Supplementary Materials and methods**

*Molecular analyses*

The mitochondrial DNA marker consisted of the first half (658 base pairs) of the COI gene of 418 *Leptidea* samples. This dataset included 236 *L. sinapis*, 61 *L. reali* and 113 *L. juvernica* specimens from various localities representative for the range of these species, and two samples each from all other congeners with available COI sequences (*L. amurensis*, *L. morsei*, *L. lactea* and *L. duponcheli*), which were used as outgroup (Table S2). The nuclear DNA marker used was the internal transcribed spacer 2 (ITS2), available for 173 *Leptidea* samples (88 *L. sinapis*, 25 *L. reali*, 56 *L. juvernica* and one sample from each outgroup; Table S2) chosen so that they are representative for most populations for which COI sequences were also available. The majority of specimens used in this study were obtained through field sampling by the authors and by collaborators from different parts of Europe (Table S2). Initial species identification was done using genitalia examinations, which allowed separation of *L. sinapis* from *L. reali* and *L. juvernica* (Dincă *et al*. 2011). Samples from Great Britain were bought from the company Worldwide Butterflies. These specimens descended from wild caught individuals from Nene Valley (Peterborough) in Great Britain. DNA was preserved by desiccation and/or by freezing the samples at -20°C until extraction.

A total of 219 already published COI DNA-sequences were obtained from the studies of (Dincă *et al*. 2011) and (Lukhtanov *et al.* 2011) (197 sequences), (Verovnik & Glogovčan 2007) (12 sequences), (Hausmann *et al*. 2011) (six sequences), (Friberg *et al*. 2008b) (one sequence), (Braby *et al*. 2006) (one sequence), (Lukhtanov *et al*. 2009) (one sequence) and (Mutanen *et al*. 2010) (one sequence), and added to the dataset. For six GenBank sequences of *L. juvernica* from Slovenia (accession numbers EF599640, EF599641, EF599643-EF599646), we have applied a correction at the beginning of the sequences, after confirmation from the submitter. Thus, the first 15 bases, originally submitted as “5'-atgttgatGAagaat-3'” became 5'-atgttgatAGagaat-3'”. The ITS2 *Leptidea* sequences available in GenBank and overlapping with our fragment (80 sequences) were used exclusively from the studies of (Dincă *et al*. 2011) and (Lukhtanov *et al.* 2011).

The DNA extraction, PCR and sequencing protocols of the samples processed in Barcelona followed the methods of Dincă *et al.* (2011) for both COI and ITS2. For the samples analyzed at Stockholm University, DNA was extracted from two legs of frozen or dried butterflies using QIAGEN’s DNeasy extraction kit (Hilden, Germany) according to the manufacturer’s protocol. The PCRs were performed in a 20 μl reaction volume using the primer pairs LCO (forward) (5'-GGTCAACAAATCATAAAGATATTGG-3') and HCO (reverse) (5'-TAAACTTCTGGATGTCCAAAAAATCA-3') (Folmer *et al*. 1994) (bp 1-658) with universal tails (see Wahlberg & Wheat 2008). The cycling profile for both primer pairs was 95 °C for five minutes followed by 40 cycles of 94 °C for 30 s, 50 °C for 30 s, 72 °C for one minute, and a final extension period of 72 °C for ten minutes. Universal forward and reverse primers (Wahlberg & Wheat 2008) were used for sequencing, which was done with a Beckman-Coulter CEQ8000 capillary sequencer.

Sequences were aligned using GENEIOUS PRO 4.7.5 (Drummond *et al*. 2009). In the case of ITS2, we left substitution heterozygotes as ambiguities. When encountering heterozygotes for insertions or deletions, we determined the phase of the sequences and edited them accordingly. For COI, the 658 bp-long alignment was ambiguity free, comprised 410 specimens of *L.* *sinapis*, *L. reali* and *L. juvernica*, and consisted exclusively of sequences longer than 620 bp. For ITS2, the 715 bp-long alignment comprised 169 sequences of *L.* *sinapis*, *L. reali* and *L. juvernica*, and consisted exclusively of sequences longer than 615 bp.

*Phylogenetic inference*

Maximum Likelihood (ML) and Bayesian Inference (BI) analyses were run for each marker separately, as well as for the combined dataset.

The Maximum Likelihood (ML) phylogenetic trees were inferred for COI, ITS2 and the combined dataset using PHYML 2.4.4 (Guindon & Gascuel 2003) implemented in GENEIOUS. The nucleotide substitution models employed were GTR+G for COI, GTR for ITS2 and GTR+G for the combined dataset based on AIC values given by jMODELTEST v 0.1 (Posada 2008) and the options available in PHYML 2.4.4. Node supports were assessed using 100 bootstrap replicates.

The Bayesian Inference (BI) analyses were run with BEAST 1.6.2 (Drummond & Rambaut 2007). The nucleotide substitution models employed were GTR+G for COI and GTR for ITS2. For the combined dataset, the BI analyses were done with the dataset partitioned by gene and with the HKY+I+G substitution model, as suggested by JMODELTEST 0.1. Base frequencies were estimated, six gamma rate categories were selected and a randomly generated initial tree was used in all cases. Parameters were estimated using two independent runs of ten million generations each and convergence was checked using the program TRACER v1.5.

Uncorrected p distances were calculated with MEGA v 5.0 (Tamura *et al*. 2011), with pair-wise deletion and including both transitions and transversions. For COI, phylogenetic analyses were based on 53 sequences, which included the 45 haplotypes inferred with TCS 1.21 plus the eight *Leptidea* outgroup sequences. For ITS2, the analyses were based on all 173 sequences available (among which four outgroup sequences), while for the combined dataset, 173 COI and ITS2 sequences were used.

*Mapping genetic divergence*

Genetic divergence for each DNA-marker (COI and ITS 2), measured by uncorrected p-distance, was mapped independently for *L. sinapis*, *L. reali* and *L. juvernica*. The only exception was the ITS2 dataset for *L. reali*, where genetic divergence was zero among all sampled localities and no figure is presented. If several sequences were available for a given locality, then the mean genetic distance between them and the other sites was used. Since the matrix of uncorrected p-distance can be affected by the presence of ambiguities or missing data in the alignment, we used only datasets that included sequences of equal lengths and without ambiguities (325 COI sequences (620 bp length) for *L. sinapis*, 60 COI sequences (658 bp length) for *L. reali*, 113 COI sequences (637 bp length) for *L. juvernica*, 74 ITS2 sequences (663 bp length) for *L. sinapis* and 52 ITS2 sequences (662 bp length) for *L. juvernica*). In each case, a matrix of uncorrected p-distances and a table of GPS coordinates (decimal degrees format) of the corresponding samples were imported in R (2.14.0) with the libraries deldir, ecodist and oce installed. We calculated a Delaunay triangulation among GPS coordinates for the collection sites. The mid points of segments composing the Delaunay triangulation were identified and the p-distance between the pair of sites composing each segment attributed to the mid-points. Mid-points and their p-distance were imported in ArcMap10 by Esri (www.esri.com), and the p-distance values interpolated through inverse distance weighting by using Spatial Analyst. This method generated a visual representation of the spatial distribution of genetic divergence with a similar procedure to that of Genetic landscapes GIS toolbox (Vandergast *et al*. 2011).

*Butterfly rearing conditions* *and courtship experiments*

The offspring of wild-collected females were reared in family groups of 3-5 individuals in 0.5L jars with *ad libitum* access to their larval host plant (*Lotus corniculatus*, *Lathyrus linifolius* or *La. pratensis*) at 23°C at either a 22h day length (inducing direct development), a 16 h day length (inducing diapause in populations from northern Europe) or at a 9 h day length (inducing diapause in populations from southern Europe). Butterflies were individualized as pupae and those set for direct development were maintained at 23°C until eclosion. Overwintering individuals were incubated outdoors, and transferred to a constant temperature room (23°C at a 22h day length) approximately a week before trials in spring. Emerging adults were sexed and individually marked with a marker pen on the ventral side of the right hind wing. Males were placed in species-specific flight cages at room temperature, whereas females were transferred into a cold room (10°C) until they were used in the experiment. When possible, in order to avoid potential effects of experiment day or time of day, we varied the species/populations that were presented to each other over the day and over the experiment period.

Each experimental trial started with a virgin female being transferred to an empty cage (0.8 x 0.8 x 0.5 m) that was located underneath 400-W metal halide lamps next to a window, letting in additional, natural daylight. The female was fed 25% sugar solution for 10 minutes, before a male was released into the cage. Courtship initiation was mediated by manipulating males and females into sitting on different cotton tipped sticks. The female was thereafter presented to the male, which often responded by climbing over to the female cotton tip, uncoiling his proboscis an initiating the display by oscillating the proboscis (see Wiklund 1977 and Friberg *et al*. 2008 for a detailed description of the courtship). As a response to the male courtship, a female can signal mating acceptance by bending her abdomen so that it becomes visible in between her wings, which facilitates copulation. Females do not signal unwillingness to mate, and unsuccessful courtships are typically terminated by the male aborting the display, and flying away (Friberg *et al*. 2008). On rare occasions a female takes off during courtship, and this behaviour is almost invariably leading to the male pursuing the female until she once again alights in the vegetation so that the male can continue his display (Wiklund 1977).

**References**

Braby MF, Vila R, Pierce N (2006) Molecular phylogeny and systematics of the Pieridae (Lepidoptera: Papilionoidea): higher classification and biogeography. *Zoological Journal of the Linnean Society*, **147**, 239-275.

Dincă V, Lukhtanov VA, Talavera G, Vila R (2011) Unexpected layers of cryptic diversity in wood white *Leptidea* butterflies. *Nature Communications*, **2**, 324.

Drummond AJ, Rambaut A (2007) BEAST: Bayesian evolutionary analysis by sampling trees. *BMC Evolutionary Biology*, **7**, 214.

Drummond AJ, Ashton B, Buxton S, Cheung M, Cooper A, Duran C, Field M, Heled J, Kearse M, Markowitz S, Moir R, Stones-Havas S, Sturrock S, Thierer T, Wilson A **(2009) Geneious v4.7. - Available from** http://www.geneious.com/

Folmer O, Black M, Hoeh W, Lutz R, Vrijenhoek R (1994) DNA primers for amplification of mitochondrial cytochrome c oxidase subunit I from diverse metazoan invertebrates.

Friberg M, Vongvanich N, Borg-Karlson A-K, Kemp DJ, Merilaita S, Wiklund C (2008) Female mate choice determines reproductive isolation between sympatric butterflies. *Behavioral Ecology and Sociobiology*, **62**, 873-886.

Fumi M (2008) Distinguishing between *Leptidea sinapis* and *L. reali* (Lepidoptera: Pieridae) using a morphometric approach: impact of measurement error on the discriminative characters. *Zootaxa*, **1819**, 40-54.

Guindon S, Gascuel O (2003) A simple, fast, and accurate algorithm to estimate large phylogenies by maximum likelihood. *Systematic Biology*, **52**, 696-704.

Hausmann A, Haszprunar G, Segerer AH, Speidel W, Behounek G, Hebert PDN (2011) Now DNA-barcoded: the butterflies and larger moths of Germany. *Spixiana*, **34**, 47-58.

Lorković, Z. 1993. *Leptidea reali* Reissinger, 1989 (= *lorkovicii* Real 1988), a new European species (Lepid., Pieridae). *Nat.* *Croatica* **2**: 1-26.

Lukhtanov VA, Dincă V, Talavera G, Vila R (2011) Unprecedented within-species chromosome number cline in the Wood White butterfly and its significance for karyotype evolution and speciation. *BMC Evolutionary Biology*, **11**, 109.

Lukhtanov VA, Sourakov A, Zakharov EV, Hebert PDN (2009) DNA barcoding Central Asian butterflies: increasing geographical dimension does not significantly reduce the success of species identification. *Molecular Ecology Resources*, **9**, 1302-1310.

Martin J-F, Gilles A, Descimon H (2003) Species concepts and sibling species: the case of *Leptidea sinapis* and *Leptidea reali.* In: *Butterflies - ecology and evolution - taking flight.* (eds Boggs CL, Watt WB, Ehrlich PR), pp. 459-476. University of Chicago Press, Chicago.

Mutanen M, Wahlberg N, Kaila L (2010) Comprehensive gene and taxon coverage elucidates radiation patterns in moths and butterflies. *Proceedings of the Royal Society of London, series B – Biological Sciences*, **277**, 2839-2848.

Posada D. (2008) jModelTest: Phylogenetic Model Averaging. *Molecular Biology and Evolution*, **25**, 1253-1256.

Réal P (1988) Lepidoptères noveaux principalement Jurassiens. Mémoires de Comité de Liaison pour les Recherches Ecofaunistiques dans le Jura. *Publication apériodique, Besançon*, 17-24.

Reissinger E (1989) Checkliste Pieride Duponchel, 1835 der Westpalaearctis (Europa, Nordwestafrika, Kaukasus, Kleinasien). *Atalanta,* **20**,149-185.

Tamura K, Peterson D, Peterson N, Stecher G, Nei M, Kumar S (2011) MEGA5: molecular evolutionary genetics analysis using maximum likelihood, evolutionary distance, and maximum parsimony methods. *Molecular Biology and Evolution*, **28**, 2731-2739.

Vandergast AG, Perry WM, Lugo RV, Hathaway SA (2011) Genetic Landscapes GIS Toolbox: tools to map patterns of genetic divergence and diversity. *Molecular Ecology Resources,* **11**, 158-161.

Verovnik R, Glogovčan P (2007) Morphological and molecular evidence of a possible hybrid zone of *Leptidea sinapis* and *L. reali* (Lepidoptera: Pieridae). *European Journal of Entomology*, **104**, 667-674.

Wahlberg N, Wheat CW (2008) Genomic outposts serve the phylogenomic pioneers: designing novel nuclear markers for genomic DNA extractions of Lepidoptera. *Systematic Biology*, **57**, 231-242.

Wiklund C (1977) Courtship behaviour in relation to female monogamy in *Leptidea sinapis* (Lepidoptera). *Oikos*, **29**, 275-283.
