# Supplementary material for: Reproductive isolation and patterns of genetic differentiation in a cryptic butterfly species complex
Source: J Evol Biol. 2013 Aug 5;26(10):2095–106. doi: 10.1111/jeb.12211 (PMC4413813; doi:10.1111/jeb.12211)
Supplement: Table S1 [file jeb0026-2095-sd2.pdf]

Table S1 List of specimens included in the study.

Sample ID	Species	COI haplotype	COI accession number	ITS2 haplotype	ITS2 accession number	Locality	Country
RVcoll.11-J537	*L. juvernica*	**hj6**	KC865984	**hj1**	KC865866	Habay-Coeuvins, Lorraine	Belgium
RVcoll.11-J547	*L. juvernica*	**hj6**	KC865985			Habay-Coeuvins, Lorraine	Belgium
RVcoll.11-J549	*L. juvernica*	**hj6**	KC865986			Habay-Coeuvins, Lorraine	Belgium
RVcoll.10-C244	*L. juvernica*	**hj1**	JF512645	**hj1**	JF512835	Príbram, Bohemia	Czech Republic
MF-11	*L. juvernica*	**hj1**	KC865979	**hj1**	KC865870	Borová, Svitavy	Czech Republic
MF-163	*L. juvernica*	**hj1**	KC865968	**hj1**	KC865872	Javorník, Hodonín	Czech Republic
MF-166	*L. juvernica*	**hj1**	KC865969	**hj1**	KC865873	Javorník, Hodonín	Czech Republic
MF-167	*L. juvernica*	**hj1**	KC865970	**hj1**	KC865874	Javorník, Hodonín	Czech Republic
MF-168	*L. juvernica*	**hj1**	KC865971	**hj1**	KC865875	Javorník, Hodonín	Czech Republic
MF-169	*L. juvernica*	**hj1**	KC865972	**hj1**	KC865876	Javorník, Hodonín	Czech Republic
RVcoll.09-X268	*L. juvernica*	**hj6**	JF512650	**hj1**	JF512782	St. Etienne, Loire	France
RVcoll.10-A259	*L. juvernica*	**hj6**	JF512651	**hj1**	JF512783	Gresse-en-Vercors, Isère	France
RVcoll.10-A262	*L. juvernica*	**hj6**	JF512652	**hj1**	JF512784	Gresse-en-Vercors, Isère	France
RVcoll.10-A263	*L. juvernica*	**hj6**	JF512653	**hj1**	JF512785	Gresse-en-Vercors, Isère	France
RVcoll.10-A264	*L. juvernica*	**hj6**	JF512654	**hj1**	KC865859	Gresse-en-Vercors, Isère	France
RVcoll.11-H425	*L. juvernica*	**hj6**	KC865983	**hj1**	KC865865	Gresse-en-Vercors, Isère	France
RVcoll.10-A269	*L. juvernica*	**hj6**	JF512655			Gresse-en-Vercors, Isère	France
MF-14	*L. juvernica*	**hj1**	KC866130	**hj1**	KC865871	Pottenstein, Bayreuth	Germany
MF-60	*L. juvernica*	**hj1**	KC865973	**hj1**	KC865877	Mainleus, Kulmbach	Germany
RVcoll.11-H238	*L. juvernica*	**hj1**	KC865961	**hj1**	KC865864	Remda, Thuringia	Germany
GenBank	*L. juvernica*	**hj8**	GU655014			Neustadt/Donau, Plattenberg, Bavaria	Germany
GenBank	*L. juvernica*	**hj1**	GU707144			Sinzing, Alpiner Steig, Bavaria	Germany
GenBank	*L. juvernica*	**hj11**	GU707187			Kallmuenz, Kirchenberg, Bavaria	Germany
RVcoll.09-X181	*L. juvernica*	**hj9**	JF512715	**hj2**	JF512788	Kilternan, Dublin	Ireland
RVcoll.09-X176	*L. juvernica*	**hj9**	JF512713	**hj2**	JF512845	Murlough, Down	Ireland
MF-34	*L. juvernica*	**hj9**	KC865989	**hj2**	KC865879	Craigavon lakes, Craigavon	Ireland
MF-35	*L. juvernica*	**hj9**	KC865990	**hj2**	KC865880	Murlough, Down	Ireland

Sample ID	Species	COI haplotype	COI accession number	ITS2 haplotype	ITS2 accession number	Locality	Country
MF-5	*L. juvernica*	**hj9**	KC865991	**hj2**	KC865881	Dring, Longford	Ireland
MF-7	*L. juvernica*	**hj9**	KC865992	**hj2**	KC865882	Raven Curracloe, Wexford	Ireland
MF-6	*L. juvernica*	**hj9**	KC866131			Kiltacky More, Clare	Ireland
RVcoll.09-X178	*L. juvernica*	**hj9**	JF512714			Milltown, Roscommon	Ireland
RVcoll.09-X183	*L. juvernica*	**hj9**	JF512716			Gortmore Point, Lough Derg, Tipperary	Ireland
RVcoll.08-R368	*L. juvernica*	**hj1**	KC865949	**hj1**	KC865858	Val di Tovo- Laghi	Italy
RVcoll.08-R369	*L. juvernica*	**hj1**	KF049418	**hj1**	KC865878	Val di Tovo- Laghi	Italy
RVcoll.06-H639	*L. juvernica*	**hj1**	JF512624	**hj1**	JF512765	Landman, Zyryanovsk	Kazakhstan
RVcoll.06-H643	*L. juvernica*	**hj1**	JF512626	**hj1**	JF512766	Landman, Zyryanovsk	Kazakhstan
RVcoll.07-Z082	*L. juvernica*	**hj7**	JF512648	**hj1**	JF512768	South Altai, Uspenka	Kazakhstan
RVcoll.07-Z081	*L. juvernica*	**hj1**	JF512627	**hj1**	JF512787	South Altai, Uspenka	Kazakhstan
RVcoll.06-H636	*L. juvernica*	**hj4**	JF512647	**hj1**	JF512846	Landman, Zyryanovsk	Kazakhstan
RVcoll.06-H634	*L. juvernica*	**hj1**	KC865948	**hj1**	KC865857	Landman, Zyryanovsk	Kazakhstan
RVcoll.11-G222	*L. juvernica*	**hj1**	KC865960	**hj1**	KF049421	Chirkain	Kazakhstan
RVcoll.06-H642	*L. juvernica*	**hj1**	JF512625			Landman, Zyryanovsk	Kazakhstan
RVcoll.11-D787	*L. juvernica*	**hj1**	KC865959			Maleevsk	Kazakhstan
RVcoll.11-G200	*L. juvernica*	**hj4**	KC865980			Narymski Mts.	Kazakhstan
RVcoll.11-G219	*L. juvernica*	**hj5**	KC865982			Balgyn	Kazakhstan
RVcoll.11-G220	*L. juvernica*	**hj4**	KC865981			Chirkain	Kazakhstan
RVcoll.07-E553	*L. juvernica*	**hj1**	HQ004601	**hj1**	JF512767	Tohanul Nou, Brașov	Romania
RVcoll.08-M325	*L. juvernica*	**hj1**	JF512573	**hj1**	JF512769	Valea Belchia, Harghita	Romania
RVcoll.09-V633	*L. juvernica*	**hj1**	JF512635	**hj1**	JF512778	Valea Belchia, Harghita	Romania
RVcoll.09-V638	*L. juvernica*	**hj2**	JF512637	**hj1**	JF512779	Valea Belchia, Harghita	Romania
RVcoll.09-V639	*L. juvernica*	**hj1**	JF512638	**hj1**	JF512780	Valea Belchia, Harghita	Romania
RVcoll.09-V641	*L. juvernica*	**hj1**	JF512639	**hj1**	JF512781	Valea Belchia, Harghita	Romania
RVcoll.08-M322	*L. juvernica*	**hj2**	HQ004596	**hj1**	JF512789	Valea Belchia, Harghita	Romania
RVcoll.08-M310	*L. juvernica*	**hj1**	HQ004600			Gheorgheni, Harghita	Romania
RVcoll.08-M311	*L. juvernica*	**hj1**	HQ004599			Gheorgheni, Harghita	Romania

Sample ID	Species	COI haplotype	COI accession number	ITS2 haplotype	ITS2 accession number	Locality	Country
RVcoll.08-M312	*L. juvernica*	**hj1**	HQ004595			Gheorgheni, Harghita	Romania
RVcoll.08-M313	*L. juvernica*	**hj1**	JF512628			Gheorgheni, Harghita	Romania
RVcoll.08-M323	*L. juvernica*	**hj1**	HQ004594			Valea Belchia, Harghita	Romania
RVcoll.08-M324	*L. juvernica*	**hj1**	HQ004597			Valea Belchia, Harghita	Romania
RVcoll.08-M331	*L. juvernica*	**hj1**	HQ004598			Gheorgheni, Harghita	Romania
RVcoll.09-V637	*L. juvernica*	**hj1**	JF512636			Valea Belchia, Harghita	Romania
RVcoll.09-V644	*L. juvernica*	**hj1**	JF512640			Valea Belchia, Harghita	Romania
RVcoll.09-V648	*L. juvernica*	**hj1**	JF512641			Valea Belchia, Harghita	Romania
RVcoll.09-V652	*L. juvernica*	**hj1**	JF512642			Valea Belchia, Harghita	Romania
RVcoll.09-V653	*L. juvernica*	**hj2**	JF512643			Valea Belchia, Harghita	Romania
RVcoll.09-V655	*L. juvernica*	**hj1**	JF512644			Valea Belchia, Harghita	Romania
RVcoll.08-Y001	*L. juvernica*	**hj1**	JF512629	**hj1**	JF512770	St. Petersburg	Russia
RVcoll.08-Y002	*L. juvernica*	**hj1**	JF512630	**hj1**	JF512771	St. Petersburg	Russia
RVcoll.08-Y006	*L. juvernica*	**hj1**	JF512632	**hj1**	JF512772	St. Petersburg	Russia
RVcoll.08-Y007	*L. juvernica*	**hj1**	JF512575	**hj1**	JF512773	Peterhof, St. Petersburg	Russia
RVcoll.08-Y009	*L. juvernica*	**hj1**	JF512633	**hj1**	JF512774	St. Petersburg	Russia
RVcoll.08-Y010	*L. juvernica*	**hj1**	JF512576	**hj1**	JF512775	Peterhof, St. Petersburg	Russia
RVcoll.08-Y011	*L. juvernica*	**hj1**	JF512634	**hj1**	JF512776	St. Petersburg	Russia
RVcoll.08-Y012	*L. juvernica*	**hj1**	JF512577	**hj1**	JF512777	Peterhof, St. Petersburg	Russia
KK09-01	*L. juvernica*	**hj3**	KC865987	**hj1**	KC865867	Kurshskaya Kosa	Russia
KK09-02	*L. juvernica*	**hj1**	KC865962	**hj1**	KC865868	Kurshskaya Kosa	Russia
KK09-03	*L. juvernica*	**hj1**	KC865963	**hj1**	KC865869	Kurshskaya Kosa	Russia
KK09-04	*L. juvernica*	**hj1**	KC865964			Kurshskaya Kosa	Russia
KK09-05	*L. juvernica*	**hj1**	KC865965			Kurshskaya Kosa	Russia
KK09-06	*L. juvernica*	**hj1**	KC865966			Kurshskaya Kosa	Russia
KK09-07	*L. juvernica*	**hj3**	KC865988			Kurshskaya Kosa	Russia
KK09-08	*L. juvernica*	**hj1**	KC865967			Kurshskaya Kosa	Russia
RVcoll.08-Y003	*L. juvernica*	**hj1**	JF512574			St. Petersburg	Russia
RVcoll.08-Y004	*L. juvernica*	**hj3**	JF512649			St. Petersburg	Russia

Sample ID	Species	COI haplotype	COI accession number	ITS2 haplotype	ITS2 accession number	Locality	Country
RVcoll.08-Y005	*L. juvernica*	**hj1**	JF512631			St. Petersburg	Russia
RVcoll.08-Y008	*L. juvernica*	**hj3**	JF512578			Peterhof, St. Petersburg	Russia
RVcoll.11-D554	*L. juvernica*	**hj1**	KC865955			Vishnevogorsk t., Chelyabinsk reg., S Ural	Russia
RVcoll.11-D555	*L. juvernica*	**hj1**	KC865956			Kirov env.	Russia
RVcoll.11-D556	*L. juvernica*	**hj1**	KC865957			Kirov env.	Russia
RVcoll.11-D557	*L. juvernica*	**hj1**	KC865958			Kirov env.	Russia
LR08-D680	*L. juvernica*	**hj1**	JF512646	**hj1**	JF512786	Jagnjenica, Radeče	Slovenia
GenBank	*L. juvernica*	**hj13**	EF599640			Vrhnika	Slovenia
GenBank	*L. juvernica*	**hj1**	EF599641			Vrhnika	Slovenia
GenBank	*L. juvernica*	**hj12**	EF599643			Barje	Slovenia
GenBank	*L. juvernica*	**hj6**	EF599644			Vrhnika	Slovenia
GenBank	*L. juvernica*	**hj11**	EF599645			Barje	Slovenia
GenBank	*L. juvernica*	**hj1**	EF599646			Barje	Slovenia
RVcoll.10-B468	*L. juvernica*	**hj1**	KC865950	**hj1**	KC865860	Kronängen	Sweden
RVcoll.10-B471	*L. juvernica*	**hj1**	KC865951	**hj1**	KF049420	Kronängen	Sweden
RVcoll.10-B479	*L. juvernica*	**hj1**	KC865952	**hj1**	KC865861	Kronängen	Sweden
RVcoll.10-B480	*L. juvernica*	**hj1**	KC865953	**hj1**	KC865862	Kronängen	Sweden
RVcoll.10-B481	*L. juvernica*	**hj1**	KC865954	**hj1**	KC865863	Kronängen	Sweden
MF-66	*L. juvernica*	**hj1**	KC865974			Kronängen	Sweden
MF-67	*L. juvernica*	**hj1**	KC865975			Kronängen	Sweden
MF-68	*L. juvernica*	**hj1**	KC865976			Kronängen	Sweden
MF-69	*L. juvernica*	**hj1**	KC865977			Kronängen	Sweden
MF-70	*L. juvernica*	**hj1**	KC865978			Kronängen	Sweden
MF-F11	*L. juvernica*	**hj1**	KC866121			Riala	Sweden
MF-F12	*L. juvernica*	**hj10**	KC866126			Riala	Sweden
MF-F5	*L. juvernica*	**hj1**	KC866122			Kronängen	Sweden
MF-F9	*L. juvernica*	**hj1**	KC866123			Kronängen	Sweden
MF-K2	*L. juvernica*	**hj1**	KC866124			Kronängen	Sweden

Sample ID	Species	COI haplotype	COI accession number	ITS2 haplotype	ITS2 accession number	Locality	Country
MF-K3	*L. juvernica*	**hj1**	KC866125			Kronängen	Sweden
RVcoll.09-T245	*L. reali*	**hr1**	JF512672	**hr1**	JF512800	Flassan, Provence	France
RVcoll.09-T250	*L. reali*	**hr1**	JF512673	**hr1**	JF512801	Flassan, Provence	France
RVcoll.09-V231	*L. reali*	**hr1**	JF512675	**hr1**	JF512802	Flassan, Provence	France
RVcoll.10-A411	*L. reali*	**hr1**	JF512607	**hr1**	JF512805	Digne les Bains, Alpes de Haute Provence	France
RVcoll.10-A412	*L. reali*	**hr1**	JF512608	**hr1**	JF512806	Digne les Bains, Alpes de Haute Provence	France
RVcoll.09-T244	*L. reali*	**hr1**	JF512671			Flassan, Provence	France
RVcoll.09-T246	*L. reali*	**hr1**	JF512604			Flassan, Provence	France
RVcoll.09-T247	*L. reali*	**hr1**	JF512605			Flassan, Provence	France
RVcoll.09-T249	*L. reali*	**hr1**	JF512606			Flassan, Provence	France
RVcoll.09-T251	*L. reali*	**hr1**	JF512674			Flassan, Provence	France
RVcoll.07-E080	*L. reali*	**hr1**	JF512658	**hr1**	JF512792	Rocca Pia, L'Aquila	Italy
RVcoll.07-E081	*L. reali*	**hr1**	JF512659	**hr1**	JF512793	Roccaraso, L'Aquila	Italy
RVcoll.07-E082	*L. reali*	**hr1**	JF512660	**hr1**	JF512794	Roccaraso, L'Aquila	Italy
RVcoll.07-E083	*L. reali*	**hr7**	JF512712	**hr1**	JF512795	Roccaraso, L'Aquila	Italy
RVcoll.10-C258	*L. reali*	**hr1**	JF512609	**hr1**	KC865946	Sant'Anatolia di Narco, Perugia	Italy
RVcoll.10-C259	*L. reali*	**hr1**	JF512610	**hr1**	KC865947	Sant'Anatolia di Narco, Perugia	Italy
RVcoll.10-C263	*L. reali*	**hr1**	JF512611			Sant'Anatolia di Narco, Perugia	Italy
RVcoll.10-C264	*L. reali*	**hr3**	JF512615			Cascia, Perugia	Italy
RVcoll.10-C268	*L. reali*	**hr1**	JF512612			Cascia, Perugia	Italy
RVcoll.10-C269	*L. reali*	**hr2**	JF512616			Cascia, Perugia	Italy
RVcoll.10-C270	*L. reali*	**hr3**	JF512704			Sibillini Mountains	Italy
RVcoll.10-C271	*L. reali*	**hr1**	JF512613			Sant'Anatolia di Narco, Perugia	Italy
RVcoll.10-C272	*L. reali*	**hr1**	JF512614			Sant'Anatolia di Narco, Perugia	Italy
RVcoll.03-H535	*L. reali*	**hr6**	JF512617	**hr1**	JF512790	Saldes, Barcelona	Spain
RVcoll.07-C613	*L. reali*	**hr1**	JF512657	**hr1**	JF512791	La Llavina, Montseny, Barcelona	Spain
RVcoll.07-W134	*L. reali*	**hr1**	JF512661	**hr1**	JF512796	Bassa d´Arres, Lleida	Spain
RVcoll.08-J396	*L. reali*	**hr1**	JF512603	**hr1**	JF512797	Viladrau, Barcelona	Spain
RVcoll.08-L090	*L. reali*	**hr1**	JF512670	**hr1**	JF512798	El Vallecillo, Aragón	Spain

Sample ID	Species	COI haplotype	COI accession number	ITS2 haplotype	ITS2 accession number	Locality	Country
RVcoll.09-T203	*L. reali*	**hr1**	JF512720	**hr1**	JF512799	Fonts del Cardener, Lleida	Spain
RVcoll.09-V713	*L. reali*	**hr1**	JF512681	**hr1**	JF512803	Yésero, Aragón	Spain
RVcoll.09-V714	*L. reali*	**hr1**	JF512682	**hr1**	JF512804	Yésero, Aragón	Spain
RVcoll.09-V320	*L. reali*	**hr1**	JF512676	**hr1**	JF512855	Meranges, Girona	Spain
RVcoll.07-W133	*L. reali*	**hr1**	KF049419	**hr1**	KC865941	Bassa d'Arrès, Lleida	Spain
RVcoll.07-W154	*L. reali*	**hr1**	GU676968	**hr1**	KC865942	Vallibierna, Aragón	Spain
RVcoll.08-H280	*L. reali*	**hr1**	KC866105	**hr1**	KC865943	Viladrau, Barcelona	Spain
RVcoll.08-R059	*L. reali*	**hr1**	GU676782	**hr1**	KC865944	La Cambreta, com. Valenciana	Spain
RVcoll.09-V321	*L. reali*	**hr1**	JF512677	**hr1**	KC865945	Meranges, Girona	Spain
MF-100	*L. reali*	**hr1**	KC866106			Montseny Mts., Barcelona	Spain
MF-101	*L. reali*	**hr1**	KC866107			Montseny Mts., Barcelona	Spain
MF-147	*L. reali*	**hr1**	KC866108			Olot, Girona	Spain
MF-150	*L. reali*	**hr1**	KC866109			Olot, Girona	Spain
MF-154	*L. reali*	**hr1**	KC866110			Banyoles, Girona	Spain
MF-90	*L. reali*	**hr5**	KC866117			Pla de la Calma, Montseny	Spain
MF-91	*L. reali*	**hr3**	KC866118			Pla de la Calma, Montseny	Spain
MF-92	*L. reali*	**hr1**	KC866111			Pla de la Calma, Montseny	Spain
MF-93	*L. reali*	**hr1**	KC866112			Pla de la Calma, Montseny	Spain
MF-95	*L. reali*	**hr1**	KC866113			Pla de la Calma, Montseny	Spain
MF-97	*L. reali*	**hr1**	KC866114			Pla de la Calma, Montseny	Spain
MF-98	*L. reali*	**hr1**	KC866115			Pla de la Calma, Montseny	Spain
MF-99	*L. reali*	**hr1**	KC866116			Pla de la Calma, Montseny	Spain
RVcoll.07-C279	*L. reali*	**hr1**	JF512656			Viladrau, Barcelona	Spain
RVcoll.07-C636	*L. reali*	**hr1**	GU675654			Meranges, Girona	Spain
RVcoll.08-H468	*L. reali*	**hr4**	GU676645			Hormiguera, Cantabria	Spain
RVcoll.08-L078	*L. reali*	**hr1**	JF512669			Vilafranca, com. Valenciana	Spain
RVcoll.09-V325	*L. reali*	**hr1**	JF512678			Meranges, Girona	Spain
RVcoll.09-V344	*L. reali*	**hr1**	JF512679			Viladrau, Barcelona	Spain
RVcoll.09-V347	*L. reali*	**hr1**	JF512680			Viladrau, Barcelona	Spain

Sample ID	Species	COI haplotype	COI accession number	ITS2 haplotype	ITS2 accession number	Locality	Country
RVcoll.09-V733	*L. reali*	**hr1**	JF512683			Valle del Tena, Aragón	Spain
RVcoll.09-V734	*L. reali*	**hr1**	JF512684			Valle del Tena, Aragón	Spain
RVcoll.09-V743	*L. reali*	**hr1**	JF512685			Valle del Tena, Aragón	Spain
RVcoll.09-V815	*L. reali*	**hr1**	JF512686			Vilallonga de Ter, Girona	Spain
GenBank	*L. sinapis*	**hs13**	HM393183			Zahmer Kaiser, Aschinger Alm, Tyrol	Austria
RVcoll.11-J546	*L. sinapis*	**hs5**	KC866023	**hs1**	KC865900	Buzenol, Lorraine	Belgium
RVcoll.11-J540	*L. sinapis*	**hs5**	KC866021			Torgny	Belgium
RVcoll.10-B385	*L. sinapis*	**hs1**	JF512693	**hs1**	JF512817	Paril village, Khadzhidimovo	Bulgaria
LR08-D577	*L. sinapis*	**hs5**	JF512721	**hs1**	JF512823	Hvoyna, Chepelare, Rodopi Mts.	Bulgaria
LR-08-D573	*L. sinapis*	**hs12**	KC866103			Hvoyna, Chepelare, Rodopi Mts.	Bulgaria
RVcoll.10-B303	*L. sinapis*	**hs5**	KC866015			Hvoyna, Chepelare, Rodopi Mts.	Bulgaria
RVcoll.10-B320	*L. sinapis*	**hs20**	KC866088			Studen Kladenets, Krumovgrad	Bulgaria
RVcoll.10-B355	*L. sinapis*	**hs5**	KC866016			Bakalite, Chernoochene	Bulgaria
RVcoll.10-B359	*L. sinapis*	**hs1**	KC866045			Bakalite, Chernoochene	Bulgaria
RVcoll.08-H769	*L. sinapis*	**hs3**	KC866005	**hs2**	KC865886	Fozzaninco	Corsica
RVcoll.08-H779	*L. sinapis*	**hs3**	KC866007	**hs2**	KC865887	N Pietroso	Corsica
RVcoll.08-R538	*L. sinapis*	**hs3**	KC866009	**hs2**	KC865888	NE Palneca	Corsica
RVcoll.08-H762	*L. sinapis*	**hs3**	KC866003			Zicavo	Corsica
RVcoll.08-H765	*L. sinapis*	**hs3**	KC866004			Zicavo	Corsica
RVcoll.08-H777	*L. sinapis*	**hs1**	KC866040			Zicavo	Corsica
RVcoll.08-H778	*L. sinapis*	**hs3**	KC866006			Col de la Vaccia	Corsica
RVcoll.08-H786	*L. sinapis*	**hs3**	KC866008			N Pietroso	Corsica
RVcoll.09-V196	*L. sinapis*	**hs3**	KC866010			SW Ghisoni	Corsica
RVcoll.09-V199	*L. sinapis*	**hs1**	KC866043			NE Palneca	Corsica
RVcoll.10-B453	*L. sinapis*	**hs1**	JF512694	**hs1**	JF512818	Žuljana, Ston, Peninsula Peljesac	Croatia
RVcoll.10-B457	*L. sinapis*	**hs5**	JF512695	**hs1**	JF512819	Pećani, Lika-Senj	Croatia
RVcoll.10-C363	*L. sinapis*	**hs8**	KC866092			Novigrad, Zadar County	Croatia
RVcoll.10-C488	*L. sinapis*	**hs1**	KC866046			Vir-Zara	Croatia
RVcoll.11-J976	*L. sinapis*	**hs5**	KC866024			Pecki/Luscani, Banovina	Croatia

**Sample ID**	**Species**	**COI haplotype**	**COI accession number**	**ITS2 haplotype**	**ITS2 accession number**	**Locality**	**Country**
RVcoll.11-J981	*L. sinapis*	**hs5**	KC866025			Ralici, Zumberak Mt.	Croatia
RVcoll.11-J988	*L. sinapis*	**hs1**	KC866052			Prevlaka, Konavle	Croatia
RVcoll.11-K008	*L. sinapis*	**hs5**	KC866026			Bedekoviceve grabe, Medjimurje	Croatia
RVcoll.10-C245	*L. sinapis*	**hs5**	JF512697	**hs1**	JF512847	Príbram, Bohemia	Czech Republic
MF-12	*L. sinapis*	**hs5**	KC866030	**hs1**	KC865913	Kromeriz, Moravia	Czech Republic
MF-164	*L. sinapis*	**hs5**	KC866032	**hs1**	KC865917	Hodonín, Hodonín	Czech Republic
MF-170	*L. sinapis*	**hs5**	KC866033	**hs1**	KC865918	Hodonín, Hodonín	Czech Republic
MF-174	*L. sinapis*	**hs16**	KC866101	**hs1**	KC865919	Hodonín, Hodonín	Czech Republic
MF-176	*L. sinapis*	**hs5**	KC866035	**hs1**	KC865935	Hodonín, Hodonín	Czech Republic
MF-10	*L. sinapis*	**hs5**	KC866133			Olomouc, Moravia	Czech Republic
MF-171	*L. sinapis*	**hs5**	KC866034			Javorník, Hodonín	Czech Republic
RVcoll.10-C248	*L. sinapis*	**hs5**	JF512698			Príbram, Bohemia	Czech Republic
RVcoll.10-A742	*L. sinapis*	**hs5**	JF512692	**hs1**	JF512816	Piusa, Polva county	Estonia
NW-JM1-14	*L. sinapis*	**hs5**	KC866128			Kaarina, Ala-Lemun kartano	Finland
RVcoll.07-E253	*L. sinapis*	**hs1**	JF512587	**hs1**	JF512811	NE Bézaudun-sur-Bine, Drôme	France
RVcoll.07-E254	*L. sinapis*	**hs7**	JF512598	**hs1**	JF512812	NE Bézaudun-sur-Bine, Drôme	France
GenBank	*L. sinapis*	**hs1**	GU828859			Cabrespine, Aude	France
MF-16	*L. sinapis*	**hs1**	KC866069			Roquefère, Aude	France
MF-17	*L. sinapis*	**hs1**	KC866135			Cabrespine, Aude	France
RVcoll.07-E249	*L. sinapis*	**hs1**	JF512585			Col de la Chaudière, Drôme	France
RVcoll.07-E250	*L. sinapis*	**hs6**	JF513034			NE Bézaudun-sur-Bine, Drôme	France
RVcoll.07-E252	*L. sinapis*	**hs1**	JF512586			NE Bézaudun-sur-Bine, Drôme	France
RVcoll.07-E255	*L. sinapis*	**hs6**	JF512599			NE Bézaudun-sur-Bine, Drôme	France
RVcoll.07-E256	*L. sinapis*	**hs6**	JF512600			NE Bézaudun-sur-Bine, Drôme	France
RVcoll.11-J544	*L. sinapis*	**hs25**	KC866097			Manosque, Alpes-de-Haute-Provence	France
RVcoll.11-J545	*L. sinapis*	**hs3**	KC866011			St.-Étienne-les-Orgues, Alpes-de-Haute-Provence	France
MF-15	*L. sinapis*	**hs5**	KC866134	**hs1**	KC865916	Pottenstein, Bayreuth	Germany
MF-36	*L. sinapis*	**hs1**	KC866129	**hs1**	KC865937	Auerbach, Deggendorf	Germany

Sample ID	Species	COI haplotype	COI accession number	ITS2 haplotype	ITS2 accession number	Locality	Country
RVcoll.11-H237	*L. sinapis*	**hs5**	KC866018	**hs1**	KC865940	Altremda, Thuringia	Germany
RVcoll.11-H239	*L. sinapis*	**hs5**	KC866019	**hs1**	KC865899	Remda, Thuringia	Germany
GenBank	*L. sinapis*	**hs19**	GU688515			Ruhpolding, Bavaria	Germany
GenBank	*L. sinapis*	**hs21**	GU688533			Lenggries Isarauen, Bavaria	Germany
MF-8	*L. sinapis*	**hs1**	KC866077			Auerbach, Deggendorf	Germany
RVcoll.11-H240	*L. sinapis*	**hs5**	KC866020			Remda, Thuringia	Germany
LR08-D094	*L. sinapis*	**hs1**	JF512708	**hs1**	JF512826	Kalavryta, Peloponessos	Greece
LR08-D203	*L. sinapis*	**hs1**	JF512709	**hs1**	JF512834	Eptalofos, Parnassos Mt.	Greece
LR08-D355	*L. sinapis*	**hs8**	KC866094	**hs1**	KC865901	Pindos Mts., Katara pass	Greece
GenBank	*L. sinapis*	**hs1**	DQ387045			Vrachneika, Peloponessos	Greece
LR-08-D389	*L. sinapis*	**hs1**	KC866080			Smolikas Mts., Pades	Greece
MF-143	*L. sinapis*	**hs18**	KC866099	**hs1**	KC865914	Budapest env.	Hungary
MF-144	*L. sinapis*	**hs5**	KC866031	**hs1**	KC865915	Budapest env.	Hungary
RVcoll.09-X180	*L. sinapis*	**hs1**	JF512691	**hs1**	JF512815	Dromore Nature Reserve, Clare	Ireland
MF-28	*L. sinapis*	**hs1**	KC866074	**hs1**	KC865922	Tully Common, Clare	Ireland
MF-30	*L. sinapis*	**hs1**	KC866075	**hs1**	KC865923	Coolorta Mullaghmore, Sligo	Ireland
MF-31	*L. sinapis*	**hs1**	KC866137	**hs1**	KC865924	The Rine, Scarriff, Clare	Ireland
MF-33	*L. sinapis*	**hs1**	KC866139	**hs1**	KC865925	Rinnamona, Clare	Ireland
RVcoll.09-X171	*L. sinapis*	**hs1**	JF512688	**hs1**	KC865896	Gortnahoon, Galway	Ireland
MF-18	*L. sinapis*	**hs1**	KC866136			Turcoughmore, Clare	Ireland
MF-32	*L. sinapis*	**hs1**	KC866138			Crossroads at Gortlecka, Clare	Ireland
RVcoll.09-X168	*L. sinapis*	**hs1**	JF512687			Bunnanagat South, Clare	Ireland
RVcoll.09-X173	*L. sinapis*	**hs1**	JF512689			Oakfield, Galway	Ireland
RVcoll.09-X174	*L. sinapis*	**hs1**	JF512690			Rockhill, Galway	Ireland
RVcoll.07-E140	*L. sinapis*	**hs3**	JF512593	**hs1**	JF512809	Borgo Val di Taro, Parma	Italy
RVcoll.07-E237	*L. sinapis*	**hs8**	JF512597	**hs1**	JF512810	Novalesa-Moncenisio, Torino	Italy
RVcoll.10-C253	*L. sinapis*	**hs8**	JF512701	**hs1**	JF512821	Monte di Malo, Veneto	Italy
RVcoll.10-C254	*L. sinapis*	**hs1**	JF512702	**hs1**	JF512822	Monte di Malo, Veneto	Italy
RVcoll.07-E217	*L. sinapis*	**hs8**	JF512596	**hs1**	JF512827	Mompantero Vecchio, Torino	Italy

Sample ID	Species	COI haplotype	COI accession number	ITS2 haplotype	ITS2 accession number	Locality	Country
RVcoll.10-C252	*L. sinapis*	**hs1**	JF512700	**hs1**	JF512830	Monte di Malo, Veneto	Italy
RVcoll.10-C251	*L. sinapis*	**hs1**	JF512699	**hs5**	JF512832	Monte di Malo, Veneto	Italy
MF-19	*L. sinapis*	**hs15**	KC866090	**hs1**	KC865920	Turin, Torino	Italy
MF-21	*L. sinapis*	**hs1**	KC866070	**hs1**	KC865938	Turin, Torino	Italy
MF-22	*L. sinapis*	**hs1**	KC866071	**hs1**	KC865921	Turin, Torino	Italy
MF-23	*L. sinapis*	**hs1**	KC866072	**hs2**	KC865889	Turin, Torino	Italy
MF-24	*L. sinapis*	**hs1**	KC866073	**hs2**	KC865936	Turin, Torino	Italy
RVcoll.08-R551	*L. sinapis*	**hs1**	KC866041	**hs1**	KC865895	Briatico, Vibo Valentia	Italy
LD-2945	*L. sinapis*	**hs1**	KC866078			Sanza, Salerno	Italy
LD-2946	*L. sinapis*	**hs1**	KC866079			Sanza, Salerno	Italy
LR-08-D800	*L. sinapis*	**hs8**	KC866095			N of Vittoria Veneto	Italy
LR-08-D801	*L. sinapis*	**hs8**	KC866096			N of Vittoria Veneto	Italy
MF-20	*L. sinapis*	**hs15**	KC866091			Turin, Torino	Italy
RVcoll.07-E138	*L. sinapis*	**hs1**	JF513011			Borgo Val di Taro, Parma	Italy
RVcoll.07-E139	*L. sinapis*	**hs3**	JF513008			Passo de Cento Croci, Parma	Italy
RVcoll.07-E141	*L. sinapis*	**hs3**	JF513009			Passo de Cento Croci, Parma	Italy
RVcoll.07-E142	*L. sinapis*	**hs3**	JF513038			Passo de Cento Croci, Parma	Italy
RVcoll.07-E173	*L. sinapis*	**hs8**	JF513033			Ozein-Visyes, Cogne Valley	Italy
RVcoll.07-E174	*L. sinapis*	**hs8**	JF512595			Ozein-Visyes, Aosta Valley	Italy
RVcoll.07-E215	*L. sinapis*	**hs3**	JF512665			Mompantero Vecchio, Torino	Italy
RVcoll.07-E216	*L. sinapis*	**hs8**	JF512666			Mompantero Vecchio, Torino	Italy
RVcoll.08-R552	*L. sinapis*	**hs1**	KC866042			Briatico, Vibo Valentia	Italy
RVcoll.10-C255	*L. sinapis*	**hs8**	JF512703			Monte di Malo, Veneto	Italy
RVcoll.10-C262	*L. sinapis*	**hs15**	KC866089			Corciano, Perugia	Italy
RVcoll.10-C276	*L. sinapis*	**hs1**	JF512705			Norcia, Perugia	Italy
RVcoll.10-C277	*L. sinapis*	**hs1**	JF512706			Norcia, Perugia	Italy
RVcoll.10-C278	*L. sinapis*	**hs1**	JF512591			Sant'Anatolia di Narco, Perugia	Italy
RVcoll.11-D491	*L. sinapis*	**hs1**	KC866047			Briatico, Vibo Valentia	Italy

Sample ID	Species	COI haplotype	COI accession number	ITS2 haplotype	ITS2 accession number	Locality	Country
RVcoll.11-I182	*L. sinapis*	**hs1**	KC866049			Pollino, Piano Ruggio	Italy
RVcoll.11-J573	*L. sinapis*	**hs1**	KC866050			Gambarie	Italy
RVcoll.11-J588	*L. sinapis*	**hs1**	KC866051			Campiglia Maritima	Italy
RVcoll.11-J850	*L. sinapis*	**hs8**	KC866093			Val d'Assa-Pedescala, Rotzo, Vicenza	Italy
RVcoll.07-Z210	*L. sinapis*	**hs2**	JF512602	**hs3**	JF512828	Saur Mts., Malyi Zhemeney	Kazakhstan
RVcoll.07-Z236	*L. sinapis*	**hs1**	JF512588	**hs3**	JF512829	Saur Mts., Malyi Zhemeney	Kazakhstan
RVcoll.06-H638	*L. sinapis*	**hs5**	JF512579	**hs3**	JF512833	Landman, Zyryanovsk	Kazakhstan
RVcoll.06-H637	*L. sinapis*	**hs2**	JF513027	**hs3**	KC865931	Landman, Zyryanovsk	Kazakhstan
RVcoll.07-Z235	*L. sinapis*	**hs1**	JF513030	**hs1**	KC865932	Saur Mts., Malyi Zhemeney	Kazakhstan
RVcoll.06-H631	*L. sinapis*	**hs11**	JF513025			Landman, Zyryanovsk	Kazakhstan
RVcoll.06-H632	*L. sinapis*	**hs12**	JF513047			Landman, Zyryanovsk	Kazakhstan
RVcoll.06-H633	*L. sinapis*	**hs5**	JF513032			Landman, Zyryanovsk	Kazakhstan
RVcoll.06-H635	*L. sinapis*	**hs5**	JF513024			Landman, Zyryanovsk	Kazakhstan
RVcoll.06-H640	*L. sinapis*	**hs1**	JF512583			Landman, Zyryanovsk	Kazakhstan
RVcoll.06-H641	*L. sinapis*	**hs8**	JF512664			Landman, Zyryanovsk	Kazakhstan
RVcoll.06-H644	*L. sinapis*	**hs5**	JF513035			Landman, Zyryanovsk	Kazakhstan
RVcoll.07-Z209	*L. sinapis*	**hs1**	JF513012			Saur Mts., Malyi Zhemeney	Kazakhstan
RVcoll.07-Z211	*L. sinapis*	**hs4**	JF513046			Saur Mts., Malyi Zhemeney	Kazakhstan
RVcoll.07-Z237	*L. sinapis*	**hs4**	JF512601			Saur Mts., Malyi Zhemeney	Kazakhstan
RVcoll.07-Z239	*L. sinapis*	**hs1**	JF513028			Saur Mts., Malyi Zhemeney	Kazakhstan
RVcoll.11-J543	*L. sinapis*	**hs5**	KC866022			Gol-Duché, Bascharage	Luxembourg
RVcoll.11-K051	*L. sinapis*	**hs1**	KC866053	**hs1**	KC865934	Rec	Macedonia
RVcoll.11-K052	*L. sinapis*	**hs5**	KC866027			Rec	Macedonia
RVcoll.11-K071	*L. sinapis*	**hs18**	KC866098			Mala Reka, Mavrovo	Macedonia
RVcoll.11-K078	*L. sinapis*	**hs22**	KC866104			Mala Reka, Mavrovo	Macedonia
RVcoll.11-K079	*L. sinapis*	**hs5**	KC866028			Mala Reka, Mavrovo	Macedonia
RVcoll.11-K080	*L. sinapis*	**hs5**	KC866029			Mala Reka, Mavrovo	Macedonia
RVcoll.06-K559	*L. sinapis*	**hs5**	JF512580	**hs1**	JF512807	Bădeni, Cluj	Romania

Sample ID	Species	COI haplotype	COI accession number	ITS2 haplotype	ITS2 accession number	Locality	Country
RVcoll.07-D500	*L. sinapis*	**hs1**	JF512584	**hs1**	JF512825	Cheile Babei, Maramureș	Romania
RVcoll.07-D962	*L. sinapis*	**hs5**	JF512581	**hs1**	JF512848	Valea Mare, Dâmbovița	Romania
RVcoll.06-K557	*L. sinapis*	**hs5**	JF513019	**hs1**	KC865890	Bădeni, Cluj	Romania
RVcoll.06-K558	*L. sinapis*	**hs1**	JF513036	**hs4**	KC865939	Bădeni, Cluj	Romania
RVcoll.07-D086	*L. sinapis*	**hs5**	JF513021	**hs1**	KC865891	Istrița Hill, Buzău	Romania
RVcoll.06-K560	*L. sinapis*	**hs5**	JF513023			Bădeni, Cluj	Romania
RVcoll.06-N005	*L. sinapis*	**hs1**	JF513018			Izvorul Mureșului, Harghita	Romania
RVcoll.07-C210	*L. sinapis*	**hs10**	JF512592			Schitul Pahomie, Vâlcea	Romania
RVcoll.07-C310	*L. sinapis*	**hs5**	KC866012			Orășeni Vale, Botoșani	Romania
RVcoll.07-D081	*L. sinapis*	**hs5**	JF513037			Istrița Hill, Buzău	Romania
RVcoll.07-D089	*L. sinapis*	**hs1**	JF513017			Istrița Hill, Buzău	Romania
RVcoll.07-D151	*L. sinapis*	**hs5**	KC866013			Dumbrăveni forest, Constanța	Romania
RVcoll.07-D475	*L. sinapis*	**hs1**	JF513029			Câțcău, Cluj	Romania
RVcoll.07-D938	*L. sinapis*	**hs9**	JF513026			Ciupercenii de Olteț, Gorj	Romania
RVcoll.07-D939	*L. sinapis*	**hs1**	JF513010			Ciupercenii de Olteț, Gorj	Romania
RVcoll.07-D940	*L. sinapis*	**hs1**	JF512662			Ciupercenii de Olteț, Gorj	Romania
RVcoll.07-E362	*L. sinapis*	**hs5**	JF512582			Pecinișca, Caraș-Severin	Romania
RVcoll.07-E366	*L. sinapis*	**hs5**	JF513020			Cerna Sat, Caraș-Severin	Romania
RVcoll.07-E367	*L. sinapis*	**hs5**	JF513039			Cerna Sat, Caraș-Severin	Romania
RVcoll.07-F511	*L. sinapis*	**hs5**	JF513022			Bădeni, Cluj	Romania
RVcoll.07-F512	*L. sinapis*	**hs1**	JF513031			Bădeni, Cluj	Romania
RVcoll.11-D553	*L. sinapis*	**hs5**	KC866017			Vishnevogorsk t., Chelyabinsk reg., S Ural	Russia
RVcoll.08-H720	*L. sinapis*	**hs23**	KC865994	**hs2**	KC865883	Gairo	Sardinia
RVcoll.08-H732	*L. sinapis*	**hs23**	KC865997	**hs2**	KC865884	South of Fonni	Sardinia
RVcoll.08-H741	*L. sinapis*	**hs23**	KC866000	**hs2**	KC865885	East of Fonni	Sardinia
RVcoll.08-H717	*L. sinapis*	**hs23**	KC865993			Gairo	Sardinia
RVcoll.08-H721	*L. sinapis*	**hs23**	KC865995			Gairo	Sardinia
RVcoll.08-H731	*L. sinapis*	**hs23**	KC865996			South of Fonni	Sardinia
RVcoll.08-H733	*L. sinapis*	**hs23**	KC865998			South of Fonni	Sardinia
RVcoll.08-H734	*L. sinapis*	**hs23**	KC865999			South of Fonni	Sardinia

Sample ID	Species	COI haplotype	COI accession number	ITS2 haplotype	ITS2 accession number	Locality	Country
RVcoll.08-H744	*L. sinapis*	**hs23**	KC866001			East of Fonni	Sardinia
LR08-D679	*L. sinapis*	**hs5**	JF512710	**hs1**	JF512824	Jagnjenica, Radeče	Slovenia
LR08-D756	*L. sinapis*	**hs5**	JF512711	**hs1**	KC865902	Račice, Ilistra Bistrica	Slovenia
GenBank	*L. sinapis*	**hs5**	EF599633			Vrhnika	Slovenia
GenBank	*L. sinapis*	**hs8**	EF599634			Tinjan	Slovenia
GenBank	*L. sinapis*	**hs1**	EF599635			Vrhnika	Slovenia
GenBank	*L. sinapis*	**hs5**	EF599636			Tinjan	Slovenia
GenBank	*L. sinapis*	**hs1**	EF599637			Barje	Slovenia
GenBank	*L. sinapis*	**hs8**	EF599638			Vrhnika	Slovenia
RVcoll.07-C470	*L. sinapis*	**hs1**	JF512623	**hs1**	JF512808	Llinars del Vallès, Barcelona	Spain
RVcoll.08-H275	*L. sinapis*	**hs1**	JF512589	**hs1**	JF512813	Viladrau, Barcelona	Spain
RVcoll.08-H281	*L. sinapis*	**hs3**	JF512594	**hs1**	JF512814	Viladrau, Barcelona	Spain
RVcoll.08-H527	*L. sinapis*	**hs3**	GU676633	**hs1**	KC865892	Gualda, La Alcarria, Guadalajara	Spain
RVcoll.08-H903	*L. sinapis*	**hs1**	JF512668	**hs1**	KC865893	La Garganta, Cáceres	Spain
RVcoll.08-R046	*L. sinapis*	**hs1**	GU676785	**hs1**	KC865894	Barranco Truchelles, Toga	Spain
RVcoll.08-R436	*L. sinapis*	**hs24**	GU675857	**hs1**	KC865933	Ames, Novais	Spain
RVcoll.11-D646	*L. sinapis*	**hs1**	KC866048	**hs1**	KC865898	Font de l'Arbre, Serra d'Aitana	Spain
GenBank	*L. sinapis*	**hs1**	AY954565			Vallgrassa, Barcelona	Spain
MF-103	*L. sinapis*	**hs1**	KC866127			Montseny Mts., Barcelona	Spain
MF-145	*L. sinapis*	**hs1**	KC866065			St. Pere de Ribes, Barcelona	Spain
MF-146	*L. sinapis*	**hs7**	KC866083			St. Pere de Ribes, Barcelona	Spain
MF-148	*L. sinapis*	**hs1**	KC866066			Banyoles, Girona	Spain
MF-149	*L. sinapis*	**hs1**	KC866067			St. Pere de Ribes, Barcelona	Spain
MF-155	*L. sinapis*	**hs17**	KC866100			Sant Celoni, Barcelona	Spain
MF-156	*L. sinapis*	**hs7**	KC866084			St. Pere de Ribes, Barcelona	Spain
MF-157	*L. sinapis*	**hs7**	KC866085			St. Pere de Ribes, Barcelona	Spain
MF-159	*L. sinapis*	**hs1**	KC866068			Montseny Mts., Barcelona	Spain
MF-160	*L. sinapis*	**hs7**	KC866086			St. Pere de Ribes, Barcelona	Spain
MF-165	*L. sinapis*	**hs7**	KC866087			Sant Celoni, Barcelona	Spain
MF-4	*L. sinapis*	**hs1**	KC866076			Olesa de Monserrat, Barcelona	Spain
RVcoll.07-C466	*L. sinapis*	**hs3**	JF512663			Cànoves, Barcelona	Spain
RVcoll.07-C467	*L. sinapis*	**hs1**	KC866119			Vallforners, Barcelona	Spain

Sample ID	Species	COI haplotype	COI accession number	ITS2 haplotype	ITS2 accession number	Locality	Country
RVcoll.08-H277	*L. sinapis*	**hs1**	JF513040			Viladrau, Barcelona	Spain
RVcoll.08-H278	*L. sinapis*	**hs3**	JF512667			Viladrau, Barcelona	Spain
RVcoll.08-H525	*L. sinapis*	**hs3**	KC866002			Gualda, La Alcarria, Guadalajara	Spain
RVcoll.08-J393	*L. sinapis*	**hs1**	JF513041			Viladrau, Barcelona	Spain
RVcoll.09-V326	*L. sinapis*	**hs1**	JF512590			Cànoves, Barcelona	Spain
RVcoll.09-V327	*L. sinapis*	**hs1**	JF513042			Vallforners, Barcelona	Spain
RVcoll.09-V328	*L. sinapis*	**hs1**	JF513043			Vallforners, Barcelona	Spain
RVcoll.09-V329	*L. sinapis*	**hs3**	JF513044			Vallforners, Barcelona	Spain
RVcoll.09-V330	*L. sinapis*	**hs1**	JF513045			Vallforners, Barcelona	Spain
RVcoll.09-V331	*L. sinapis*	**hs1**	JF513013			Vallforners, Barcelona	Spain
RVcoll.09-V341	*L. sinapis*	**hs1**	JF513014			Viladrau, Barcelona	Spain
RVcoll.09-V342	*L. sinapis*	**hs1**	JF513015			Viladrau, Barcelona	Spain
RVcoll.09-V343	*L. sinapis*	**hs1**	KC866132			Viladrau, Barcelona	Spain
RVcoll.09-V345	*L. sinapis*	**hs1**	JF513016			Viladrau, Barcelona	Spain
RVcoll.09-X553	*L. sinapis*	**hs7**	KC866081			2 km SW Uribarri, País Vasco	Spain
RVcoll.09-X562	*L. sinapis*	**hs7**	KC866082			Sorauren, Navarra	Spain
RVcoll.10-A017	*L. sinapis*	**hs1**	KC866044			Cuevas del Valle, Ávila	Spain
MF-61	*L. sinapis*	**hs5**	KC866036	**hs1**	KC865926	Riala	Sweden
MF-62	*L. sinapis*	**hs5**	KC866037	**hs1**	KC865927	Riala	Sweden
MF-63	*L. sinapis*	**hs14**	KC866102	**hs1**	KC865928	Riala	Sweden
MF-64	*L. sinapis*	**hs5**	KC866038	**hs1**	KC865929	Riala	Sweden
MF-65	*L. sinapis*	**hs5**	KC866039	**hs1**	KC865930	Riala	Sweden
RVcoll.10-A291	*L. sinapis*	**hs5**	KC866014	**hs1**	KC865897	Riala	Sweden
160410QT77	*L. sinapis*	**hs7**	JF512707	**hs1**	JF512831	Gampel Bratsch, Martigny, Valais	Switzerland
MF-118	*L. sinapis*	**hs1**	KC866054	**hs1**	KC865903	Castor, Nene Valley, Peterborough	UK
MF-119	*L. sinapis*	**hs1**	KC866055	**hs1**	KC865904	Castor, Nene Valley, Peterborough	UK
MF-120	*L. sinapis*	**hs1**	KC866056	**hs1**	KC865905	Castor, Nene Valley, Peterborough	UK
MF-121	*L. sinapis*	**hs1**	KC866057	**hs1**	KC865906	Castor, Nene Valley, Peterborough	UK
MF-122	*L. sinapis*	**hs1**	KC866058	**hs1**	KC865907	Castor, Nene Valley, Peterborough	UK
MF-123	*L. sinapis*	**hs1**	KC866059	**hs1**	KC865908	Castor, Nene Valley, Peterborough	UK
MF-124	*L. sinapis*	**hs1**	KC866060	**hs1**	KC865909	Castor, Nene Valley, Peterborough	UK
MF-125	*L. sinapis*	**hs1**	KC866061	**hs1**	KC865910	Castor, Nene Valley, Peterborough	UK

Sample ID	Species	COI haplotype	COI accession number	ITS2 haplotype	ITS2 accession number	Locality	Country
MF-126	*L. sinapis*	**hs1**	KC866062	**hs1**	KC865911	Castor, Nene Valley, Peterborough	UK
MF-127	*L. sinapis*	**hs1**	KC866063	**hs1**	KC865912	Castor, Nene Valley, Peterborough	UK
MF-128	*L. sinapis*	**hs1**	KC866064			Castor, Nene Valley, Peterborough	UK
RVcoll.10-C197	*L. sinapis*	**hs5**	JF512696	**hs1**	JF512820	Novaya Ilienko station, Derkul river, Luhansk reg.	Ukraine
Nz091	*L. amurensis*		JF512621			Bulgan	central Mongolia
RVcoll.10-C186	*L. amurensis*		JF512622		JF512841	Jiexiu county, Shanxi	China
RVcoll.09-V207	*L. duponcheli*		JF512569		JF512852	Oraison, Alpes de Haute Provence	France
MF-1	*L. duponcheli*		KC866120			Skopje	Macedonia
RVcoll.10-C189	*L. lactea*		JF512717		JF512849	Qin Ling Shan, Madao, Liuba county, Shaanxi	China
RVcoll.10-C195	*L. lactea*		JF512718			Qin Ling Shan, Zhouzhi (Erqu) county, Shaanxi	China
RVcoll.07-Z124	*L. morsei*		JF512618			South Altai, Markakol	Kazakhstan
RVcoll.08-M498	*L. morsei*		HQ004591		JF512840	Bădeni, Cluj	Romania
