# Supplementary material for: Reproductive isolation and patterns of genetic differentiation in a cryptic butterfly species complex
Source: J Evol Biol. 2013 Aug 5;26(10):2095–106. doi: 10.1111/jeb.12211 (PMC4413813; doi:10.1111/jeb.12211)
Supplement: Figure S1–S5 [file jeb0026-2095-sd3.pdf]

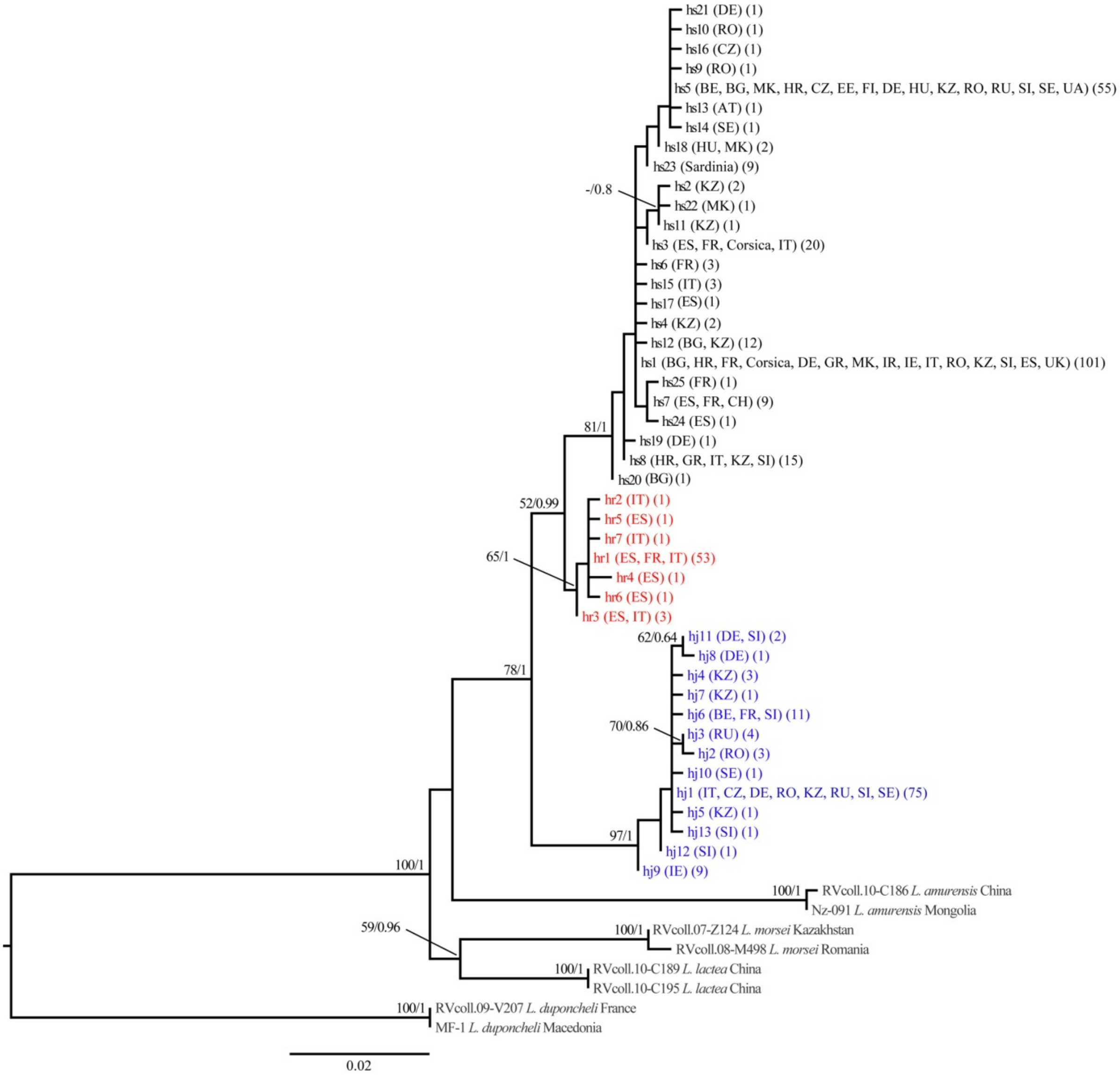

Fig. S1 Maximum likelihood tree of mitochondrial COI haplotypes of *L. sinapis* (black text), *L. reali* (red text) and *L. juvernica* (blue text). Two specimens per species of *L. amurensis*, *L. lactea*, *L. morsei* and *L. duponcheli* were used as outgroup. Maximum Likelihood bootstrap supports (≥ 50) and Bayesian posterior probabilities (≥ 0.5) are shown next to recovered nodes. Countries (abbreviated) and numbers of specimens corresponding to each haplotype are indicated in parentheses.

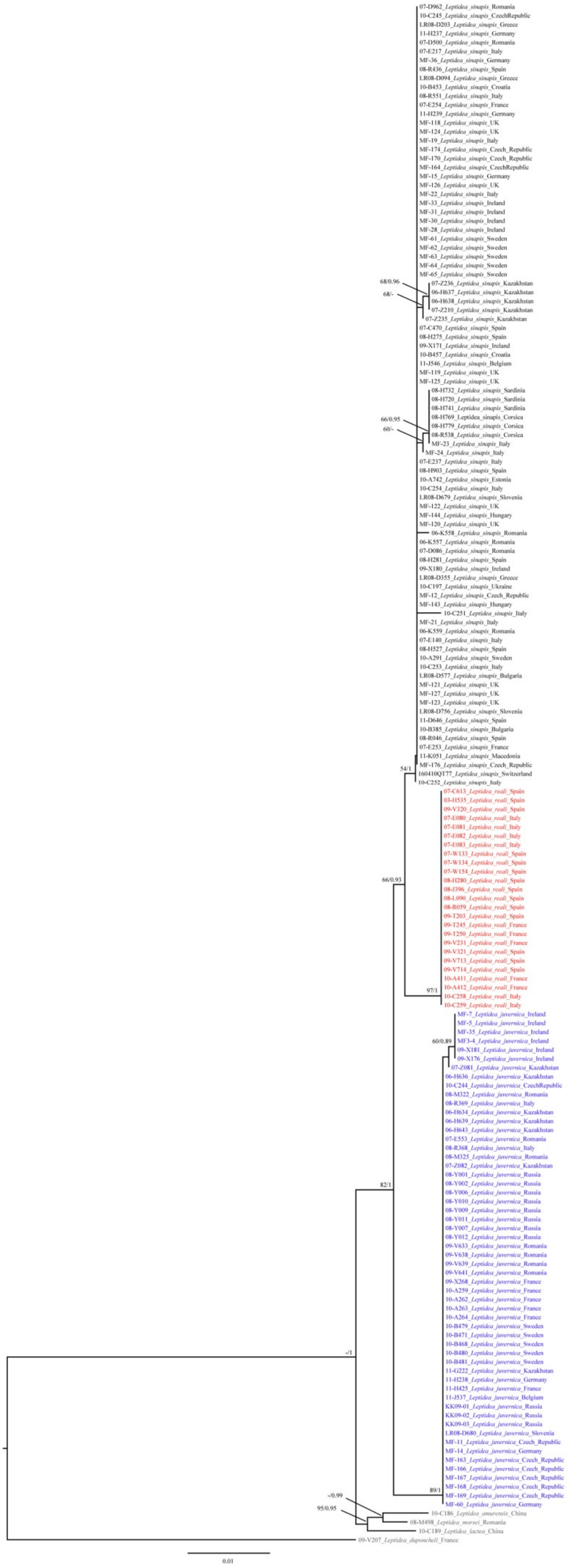

Fig. S2 Maximum likelihood tree of nuclear ITS2 sequences of *L. sinapis* (black text), *L. reali* (red text) and *L. juvernica* (blue text). One specimen per species of *L. amurensis*, *L. lactea*, *L. morsei* and *L. duponcheli* was used as outgroup. Maximum Likelihood bootstrap supports (≥ 50) and Bayesian posterior probabilities (≥ 0.5) are shown next to recovered nodes.

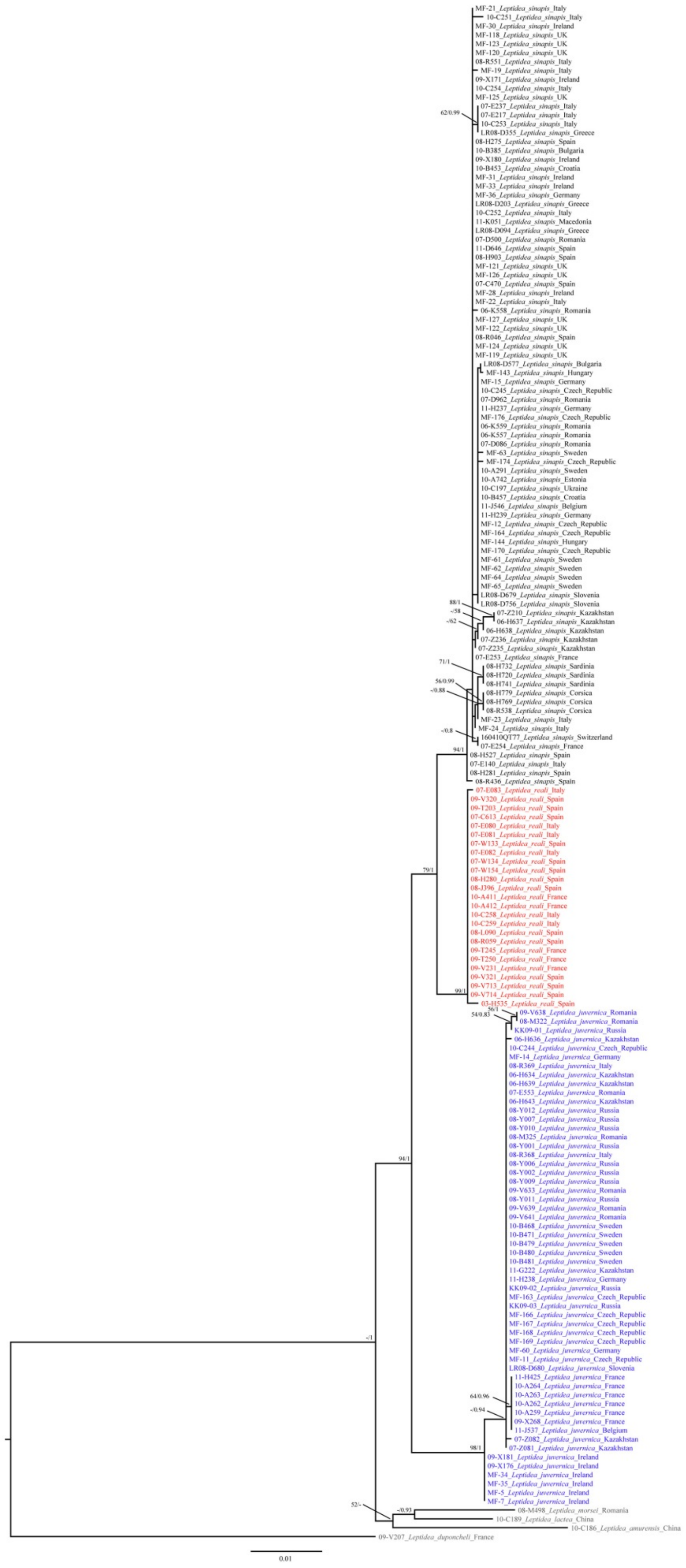

Fig. S3 Maximum likelihood tree of the combined dataset of mitochondrial COI and nuclear ITS2 sequences of *L. sinapis* (black text), *L. reali* (red text) and *L. juvernica* (blue text). One specimen per species of *L. amurensis*, *L. lactea*, *L. morsei* and *L. duponcheli* was used as outgroup. Maximum Likelihood bootstrap supports (≥ 50) and Bayesian posterior probabilities (≥ 0.5) are shown next to recovered nodes.

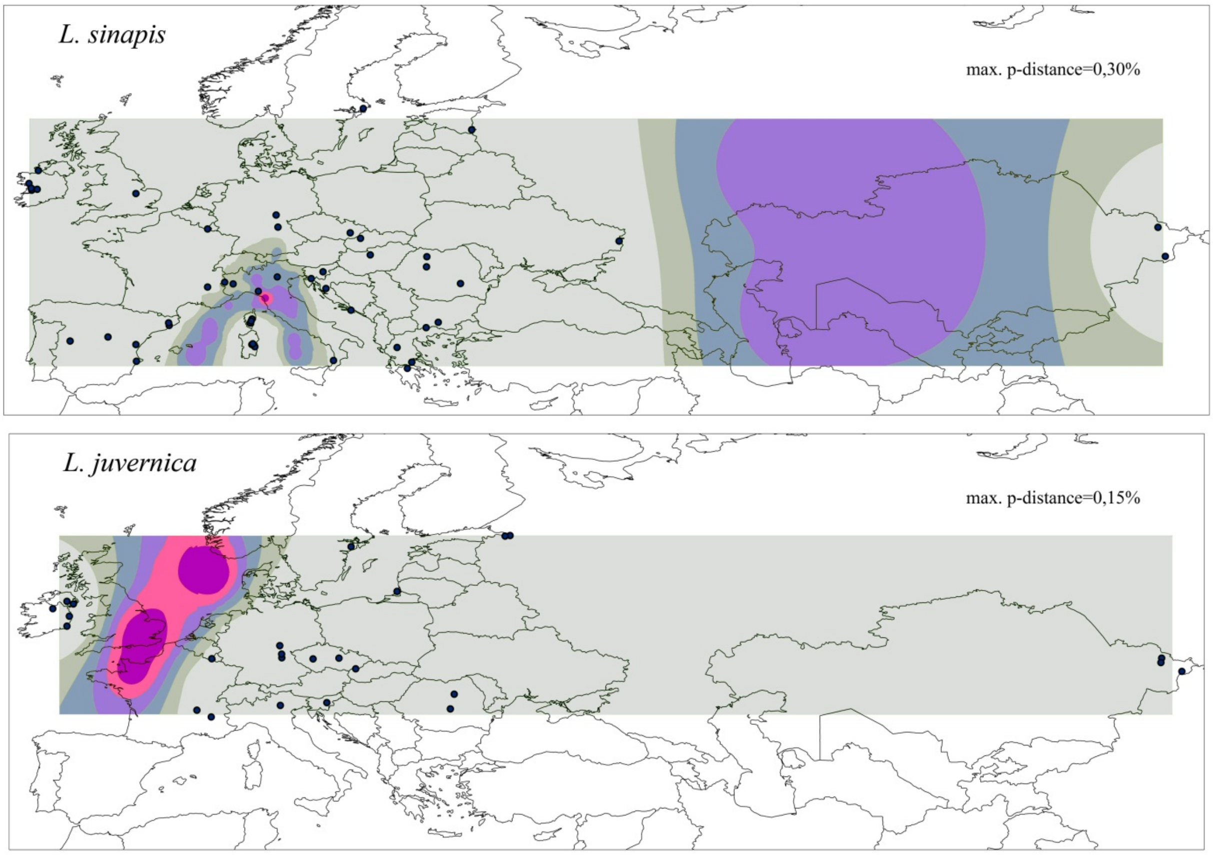

Fig. S4 Maps of genetic divergence for *L. sinapis* and *L. juvernica* based on ITS2 uncorrected p-distance. The colours indicate increasing levels of genetic divergence in the following order: light grey, grey, light blue, light violet, pink and violet. All colours indicate geographical areas situated midway between different haplotypes. No map of ITS2-based genetic divergence was built for *L. reali* since all sequences were identical.

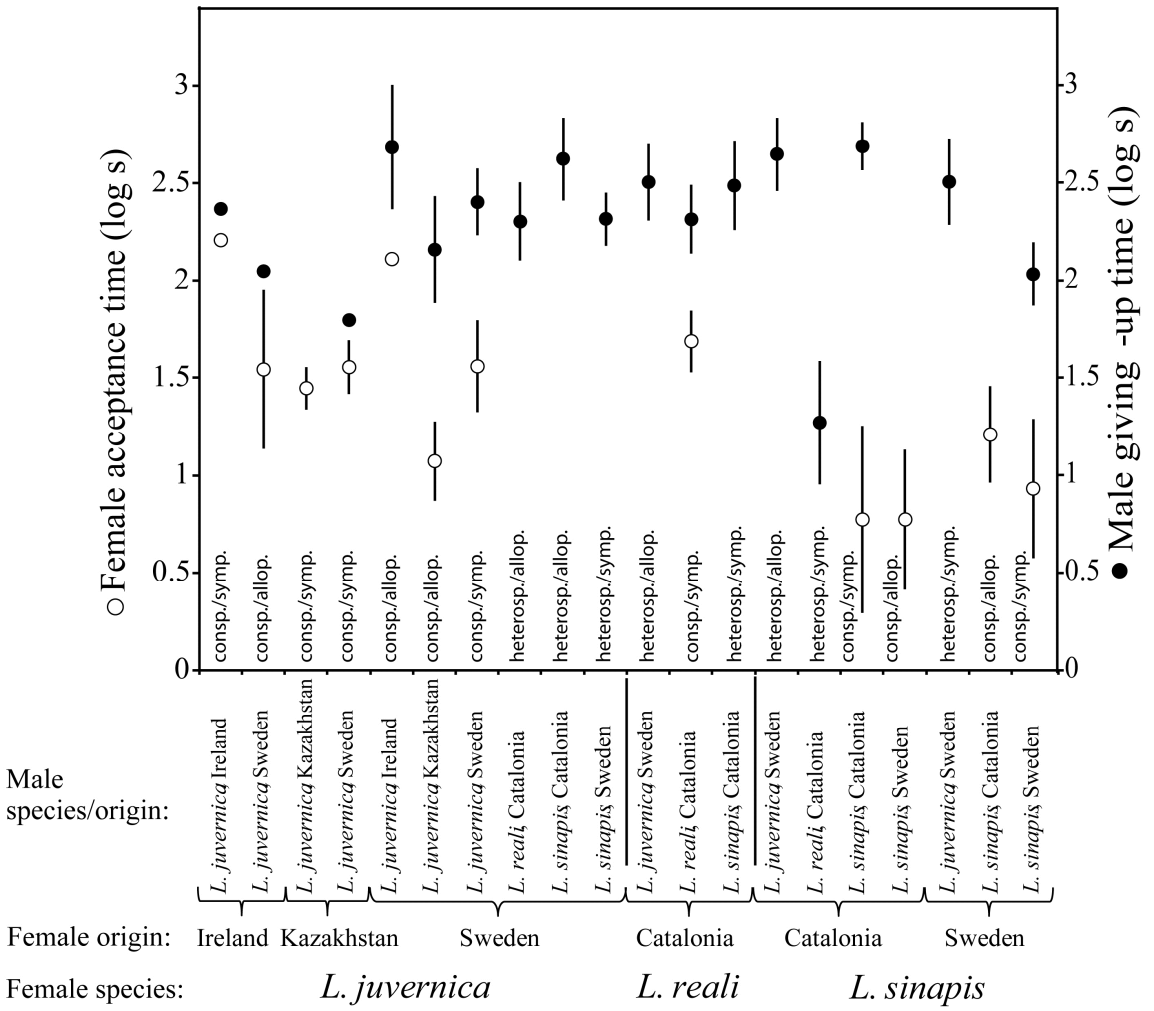

Fig. S5 Female acceptance times (open circles; log s ± 95% confidence intervals) and the male giving up times (filled circles; log s ± 95% confidence intervals) in the different interactions. It is also indicated whether the male-female interaction was heterospecific, and whether the interacting individuals descended from allopatric or sympatric populations.
